# OmiEmbed: A Unified Multi-Task Deep Learning Framework for Multi-Omics Data

**DOI:** 10.3390/cancers13123047

**Published:** 2021-06-18

**Authors:** Xiaoyu Zhang, Yuting Xing, Kai Sun, Yike Guo

**Affiliations:** 1 Data Science Institute, Imperial College London, London SW7 2AZ, UK; y.xing19@imperial.ac.uk (Y.X.); k.sun@imperial.ac.uk (K.S.); 2 Department of Computer Science, Hong Kong Baptist University, Hong Kong 999077, China

**Keywords:** multi-omics data, deep learning, multi-task learning, survival prediction, cancer classification

## Abstract

**Simple Summary:**

OmiEmbed is a unified multi-task deep learning framework for multi-omics data, supporting dimensionality reduction, multi-omics integration, tumour type classification, phenotypic feature reconstruction and survival prediction. The framework is comprised of deep embedding and downstream task modules to capture biomedical information from high-dimensional omics data. OmiEmbed outperformed state-of-the-art methods on all three types of downstream tasks: classification, regression and survival prediction. Better performance was achieved using the multi-task training strategy compared to training each downstream task individually. With the multi-task strategy, OmiEmbed learnt a comprehensive omics embedding containing information from multiple tasks. OmiEmbed is open source, well-organised and convenient to be extended to other customised input data, network structures and downstream tasks, which has promising potential to facilitate more accurate and personalised clinical decision making.

**Abstract:**

High-dimensional omics data contain intrinsic biomedical information that is crucial for personalised medicine. Nevertheless, it is challenging to capture them from the genome-wide data, due to the large number of molecular features and small number of available samples, which is also called “the curse of dimensionality” in machine learning. To tackle this problem and pave the way for machine learning-aided precision medicine, we proposed a unified multi-task deep learning framework named OmiEmbed to capture biomedical information from high-dimensional omics data with the deep embedding and downstream task modules. The deep embedding module learnt an omics embedding that mapped multiple omics data types into a latent space with lower dimensionality. Based on the new representation of multi-omics data, different downstream task modules were trained simultaneously and efficiently with the multi-task strategy to predict the comprehensive phenotype profile of each sample. OmiEmbed supports multiple tasks for omics data including dimensionality reduction, tumour type classification, multi-omics integration, demographic and clinical feature reconstruction, and survival prediction. The framework outperformed other methods on all three types of downstream tasks and achieved better performance with the multi-task strategy compared to training them individually. OmiEmbed is a powerful and unified framework that can be widely adapted to various applications of high-dimensional omics data and has great potential to facilitate more accurate and personalised clinical decision making.

## 1. Introduction

With the increasingly massive amount of omics data generated from emerging high-throughput technologies, the large-scale, cost-efficient and comprehensive analysis of biological molecules becomes an everyday methodology for biomedical researchers [1,2]. The analysis and assessment of different types of omics data facilitate the integration of molecular features during the standard diagnostic procedure. For instance, in the 2016 World Health Organization (WHO) classification of central nervous system (CNS) tumours [3], an integrative method combining both histopathology and molecular information was recommended for the identification of multiple tumour entities. Nevertheless, most of these molecular features designed to aid diagnosis are manually selected biomarkers referring to specific genetic alterations, which neglects the genome-wide patterns correlated with disease prognosis and other phenotypic outcomes. In recent years, instead of focusing on the effect of specific molecular features, many researchers began to delve into the overall picture of genome-wide omics data and try to obtain the deep understanding of diseases and uncover crucial diagnostic or prognostic information from it [4,5,6,7].

It is challenging to analyse genome-wide high dimensional omics data because of the mismatch between the number of molecular features and the number of samples. The dimensionality of genome-wide omics data is fairly high. For example, a RNA-Seq gene expression profile consists of more than 60,000 identifiers, and a HumanMethylation450 (450 K) DNA methylation profile has more than 485,000 probes, while the number of available samples in an omics dataset is normally small, due to the difficulty of patient recruitment and sample collection. This phenomenon is called “the curse of dimensionality” in machine learning, which would cause the massive overfitting of a model and make samples hard to cluster [8]. To overcome this issue, the number of molecular features used in downstream tasks is required to decrease significantly. Two common approaches are (1) to manually select a subset of the molecular features related to the downstream task based on domain knowledge; (2) to apply traditional dimensionality reduction algorithms, e.g., principal component analysis (PCA).

Inspired by the significant success in fields like computer vision [9] and natural language processing [10], deep learning approaches have been applied to analyse the complicated and nonlinear relationships between molecular features of high-dimensional omics data and detect genome-wide biological patterns from them [11,12,13]. With the deep learning mechanism, molecular features can be automatically selected during the training process without manual selection. Multiple downstream tasks were conducted on different types of high-dimensional omics data, including dimensionality reduction [11,14], disease type classification [6,15], survival prediction [4,16]. However, there is no unified deep learning method, to the best of our knowledge, that can simultaneously conduct all aforementioned downstream tasks together on any combination of omics types.

Here, we proposed a unified multi-task deep learning framework named OmiEmbed for integrated multi-omics analysis. The OmiEmbed framework is comprised of two main components: deep embedding module and downstream task module. In the deep embedding module, high-dimensional multi-omics data were embedded into a low-dimensional latent space to tackle the challenge of “dimensionality curse”. The learnt novel representation of each sample was then fed to multiple downstream networks, which were trained simultaneously with a joint loss function and the multi-task training strategy. Different downstream tasks that were already implemented in OmiEmbed include tumour type classification, demographic and clinical feature (e.g., age, gender, primary site, and disease stage of sample) reconstruction and prognosis prediction (i.e., predicting the survival function of each input sample). The model was trained and evaluated on two publicly available omics datasets, the Genomic Data Commons (GDC) pan-cancer multi-omics dataset [17] and the GSE109381 brain tumour methylation (BTM) dataset [5]. Our model achieved promising results for all aforementioned downstream tasks and outperformed other corresponding existing methods. With the multi-task training strategy, OmiEmbed was able to infer all downstream tasks simultaneously and efficiently. Better results were achieved in the multi-task scenario, compared to training and inferring each downstream task separately.

## 2. Related Work

The representation learning ability of DNNs (deep neural networks) has been widely verified by the significant breakthrough in computer vision and natural language processing. Inspired by this achievement, a number of deep learning approaches have been applied to high-dimensional omics data for different downstream tasks in recent years.

The most common downstream task is classification. Danaee et al. [18] presented a cancer detection model that discriminated breast tumour samples from normal samples using gene expression data based on a stacked denoising autoencoder (SDAE). Lyu and Haque [19] reshaped the high-dimensional RNA-Seq data into images and applied a convolutional neural network (CNN) for tumour type classification on the GDC dataset, which obtained an accuracy of 95.59%. Rhee et al. [20] proposed a hybrid model that was comprised of a graph convolution neural network (GCN) and a relation network (RN) for breast tumour subtype classification using gene expression data and protein–protein interaction (PPI) networks. Jurmeister et al. [21] developed a multi-layer neural network to distinguish metastatic head and neck squamous cell carcinoma (HNSC) from primary squamous cell carcinoma of the lung (LUSC), with an accuracy of 96.4% in the validation cohort. The AffinityNet [15] was a data efficient deep learning model that comprised multiple stacked K-nearest neighbours (KNN) attention pooling layers for tumour type classification. OmiVAE [6] was an end-to-end deep learning method designed for tumour type classification, based on a deep generative model variational autoencoder (VAE), achieving an accuracy of 97.49% among 33 tumour types and the normal control using gene expression and DNA methylation data from the GDC dataset.

Another typical task that has been tackled by deep learning approaches recently is the prediction of prognosis status from high-dimensional omics data. Chaudhary et al. [4] applied a vanilla autoencoder (AE) to reduce the dimensionality of multi-omics data which was comprised of gene expression, DNA methylation and miRNA expression profiles, and used the learnt representation to identify two different survival subgroups of liver tumours by Cox proportional hazard model (CoxPH), K-means clustering and support vector machine (SVM). In their experiment, a concordance index (C-index) of 0.68 was achieved on the liver tumour subjects from the GDC dataset. The deep learning model applied in this research was not an end-to-end model, and the embedding learnt by the network was used separately outside the network for downstream tasks. Huang et al. [22] implemented a deep learning network with the CoxPH model to predict prognosis for breast tumour using multi-omics data, cancer biomarkers and a gene co-expression network. The aforementioned research focused mostly on tumour samples of specific primary site and neglected the information cross different tumour types which had the potential to improve the performance of survival prediction for each tumour type. Cheerla and Gevaert [16] constructed a multimodal deep learning network to predict the survival of subjects for 20 different tumour types in the GDC dataset which achieved an overall C-index of 0.78 based on additional clinical information and histopathology whole slide images (WSIs) besides the multi-omics data.

There are also several attempts on applying deep learning methodology to multiple downstream tasks for high-dimensional omics data. Amodio et al. [7] presented a deep neural network method named SAUCIE to explore single-cell gene expression data and perform multiple data analysis tasks, including clustering, batch correction, imputation and denoising, and visualisation. However, the backbone of SAUCIE was basically an autoencoder used for embedding learning, and most of the downstream tasks were required to conduct outside the network separately, hence the network was not able to perform all of the tasks simultaneously with a single training process. Deepathology [23] was another deep learning method for omics data analysis which adopted the idea of multi-task learning. This model encoded gene expression profile into a low-dimensional latent vector to predict the tumour type and primary site of the input sample, which obtained an accuracy of 98.1% for primary site prediction and 95.2% for tumour type classification. In spite of the good results on multiple classification tasks, deepathology was not able to perform the more complicated survival prediction task and did not adopt any state-of-the-art deep multi-task learning optimisation mechanism.

## 3. Materials and Methods

### 3.1. Datasets

Two publicly available datasets were used as examples to demonstrate the ability of OmiEmbed: the Genomic Data Commons (GDC) pan-cancer multi-omics dataset [17] and the DNA methylation dataset of human central nervous system tumours (GSE109381) [5]. The overview information of the two datasets was summarised in Table 1.

The GDC pan-cancer dataset is one of the most comprehensive and widely used multi-omics dataset. It comprises high-dimensional omics data and corresponding phenotype data from two cancer genome programmes: The Cancer Genome Atlas (TCGA) [24] and Therapeutically Applicable Research to Generate Effective Treatment (TARGET). The TARGET programme mainly focuses on pediatric cancers. Three types of omics data from the GDC dataset were used in our experiments, including RNA-Seq gene expression profiling, DNA methylation profiling and miRNA expression profiling. The dimensionalities of the three types of omics data are 60,483, 485,577 and 1881 respectively. This dataset consists of 36 different types of tumour samples, along with corresponding normal control samples, among which 33 tumour types are from TCGA and 3 tumour types are from TARGET. The detailed tumour type information was tabulated in Supplementary Table S1. A wide range of phenotype features are also available in the GDC dataset including demographics (e.g., age and gender), clinical sample information (e.g., primary site and disease stage of the sample) and the survival information (recorded time of death or censoring).

The GSE109381 brain tumour methylation (BTM) dataset from the Gene Expression Omnibus (GEO) is one of the largest DNA methylation datasets specifically targeting brain tumours. We integrated both the reference set and validation set of this dataset and the whole dataset consists of 3905 samples, with almost all WHO-defined central nervous system (CNS) tumour entities [3] and eight non-neoplastic control CNS regions. The genome-wide DNA methylation profile for each sample was generated using Infinium HumanMethylation450 BeadChip (450 K) arrays, which is the same platform used for the GDC DNA methylation data. Each sample in this dataset has two types of diagnostic label, the histopathological class label defined by the latest 2016 WHO classification of CNS tumours [3] and the methylation class label defined by the original paper of this dataset [5]. The detailed tumour type information of the two label systems was listed in Supplementary Tables S2 and S3. Other phenotypic information is also available in this dataset, including age, gender and the disease stage of each sample.

### 3.2. Omics Data Process

Raw data of the GSE109381 BTM dataset downloaded from GEO (https://www.ncbi.nlm.nih.gov/geo/query/acc.cgi?acc=GSE109381, accessed on 30 August 2019) were first processed by the Bioconductor R package minfi [25] to obtain the beta value of each CpG probe. Beta value is the ratio of methylated signal intensities and the overall signal intensities, which indicates the methylation level of a specific CpG site. The DNA methylation profile generated by the 450 K array has 485,577 probes in total. Certain probes were removed during the feature filtering step according to the following criteria: probes targeting the Y chromosome (*n* = 416), probes containing the dbSNP132Common single-nucleotide polymorphism (SNP) (*n* = 7998), probes not mapping to the human reference genome (hg19) uniquely (one mismatch allowed) (*n* = 3965), probes not included in the latest Infinium MethylationEPIC BeadChip (EPIC) array (*n* = 32,260), the SNP assay probes (*n* = 65), the non-CpG loci probes (*n* = 3091) and probes with missing values (N/A) in more than 10% of samples (*n* = 2). We followed some of the criteria mentioned in the original paper of this dataset [5]. Overall, 46,746 probes were filtered out, which results in a final DNA methylation feature set of 438,831 CpG sites.

For the GDC pan-cancer dataset, the harmonised data of three omics types were downloaded from the UCSC Xena data portal (https://xenabrowser.net/datapages/, accessed on 1 May 2019) with the original data dimensionality. Each RNA-Seq gene expression profile is comprised of 60,483 identifiers referring to corresponding genes. The expression level is quantified by the fragments per kilobase of transcript per million mapped reads (FPKM) value, which has been $\log_{2}$-transformed. Feature filtering was applied to the gene expression data: targeting Y chromosome (*n* = 594) and zero expression in all samples (*n* = 1904). In total, 2440 genes were removed, leaving 58,043 molecular features for further analyses. As for miRNA expression profiles, the expression level of each miRNA stem-loop identifier was measured by the $\log_{2}$-transformed reads per million mapped reads (RPM) value. All of the miRNA identifiers were kept in our experiments. For both the gene expression and miRNA expression profiles, the expression values were normalised to the range of 0 to 1 due to the input requirement of the OmiEmbed framework. The DNA methylation data in the GDC dataset were filtered based on the same criteria used for the BTM dataset. The remaining missing values in all datasets mentioned above were imputed by the mean of corresponding molecular features.

### 3.3. Overall Architecture

**Figure 1:**
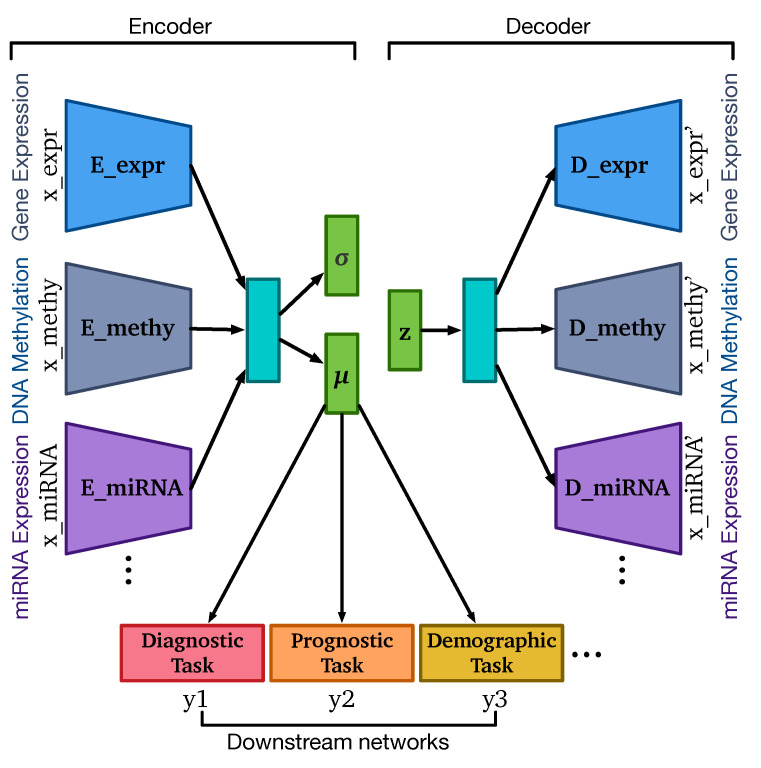
The overall architecture of OmiEmbed is comprised of two main components: the VAE deep embedding networks and the downstream task networks. The number of omics types and downstream tasks can be modified based on the user needs and requirements of the experiment. E_expr, E_methy and E_miRNA represent encoders of gene expression, DNA methylation and miRNA expression respectively. Similarly, D_expr, D_methy and D_miRNA represent decoders of gene expression, DNA methylation and miRNA expression. μ, σ and z represent the mean vector, the standard deviation vector and the latent vector calculated by the reparameterisation trick, respectively.

OmiEmbed is a unified end-to-end multi-view multi-task deep learning framework designed for high-dimensional multi-omics data, with application to dimensionality reduction, tumour type classification, multi-omics integration, demographic and clinical feature reconstruction, and survival prediction. The overall architecture of OmiEmbed is comprised of a deep embedding module and one or multiple downstream task modules, which is illustrated in Figure 1.

The role of the deep embedding module in OmiEmbed is to embed high-dimensional multi-omics profiles into a low-dimensional latent space for the downstream task modules. The backbone network that we used in the deep embedding module is variational autoencoder (VAE) [26]. VAE is a deep generative model which is also effective to capture the data manifold from high-dimensional data. We assume each sample $x^{\left(i\right)}\in R^{d}$ in the multi-omics dataset D can be represented by, and generated from, a latent vector $z^{\left(i\right)}\in R^{p}$, where p≪d. In the generation process, each latent vector is first sampled from a prior distribution $p_{\theta }\left(z\right)$, and then the multi-omics data of each sample are generated from the conditional distribution $p_{\theta }\left(x|z\right)$, where θ is the set of learnable parameters of the decoder. In order to address the intractability of the true posterior $p_{\theta }\left(z|x\right)$, the variational distribution $q_{\phi }\left(z|x\right)$ is introduced to approximate $p_{\theta }\left(z|x\right)$, where ϕ is the set of learnable parameters of the encoder. As a result, the VAE network is optimised by maximizing the variational lower bound, formularised as below:

(1) $$E_{z∼q_{\phi }\left(z|x\right)}\log p_{\theta }\left(x|z\right)-D_{\mathrm{KL}}\left(q_{\phi }\left(z|x\right)∥p_{\theta }\left(z\right)\right)$$

where $D_{\mathrm{KL}}$ is the Kullback–Leibler (KL) divergence between two probability distributions [8].

We applied the framework of VAE to our deep embedding module to obtain the low-dimensional latent vector that can represent the original high-dimensional omics data in the downstream task modules. For each type of omics data, the input profiles were first encoded into corresponding vectors with specific encoders. Those vectors of different omics types were then concatenated together in the subsequent hidden layer and encoded into one multi-omics vector. Based on the idea of VAE, the multi-omics vector was connected to two bottleneck layers in order to obtain the mean vector μ and the standard deviation vector σ. These two vectors defined the Gaussian distribution $N\left(\mu ,\sigma \right)$ of the latent variable z given the input sample x, which is the variational distribution $q_{\phi }\left(z|x\right)$. Since sampling z from the learnt distribution is not differentiable and suitable for backpropagation, the reparameterisation trick is applied as follows:

(2) z=μ+σϵ

where ϵ is a random variable sampled from the standard normal distribution N(0,I). The latent variable z was then fed to the decoders with a symmetrical network structure to obtain the reconstructed multi-omics data $x^{\prime }$.

We provided two types of detailed network structure for the encoders and decoders in the deep embedding module, the one-dimensional convolutional neural network (CNN) and the fully connected neural network (FC). The network structures of the two types of deep embedding modules were illustrated in Supplementary Figures S1 and S2. Both network types shared the same architecture, and other state-of-the-art or customised embedding networks can be easily added to the OmiEmbed framework with minimal modification using our open-source repository in Github (https://github.com/zhangxiaoyu11/OmiEmbed/, accessed on 19 May 2021). With the deep embedding module, we can obtain the low-dimensional representation of the input omics data. This new representation can directly replace the original omics data as the input of any downstream task. For instance, when the latent dimension is set to 2 or 3, the new representation can be used for visualisation purpose. Nevertheless, we can also attach one or multiple downstream task networks to the bottleneck layer of the deep embedding module to obtain an end-to-end multi-task model, which is able to guide the embedding step with objectives and share information among different tasks.

Three main types of end-to-end downstream tasks were provided in the OmiEmbed framework: classification task, regression task and survival prediction task. Each downstream task fell into one of these categories can be trained individually or collaboratively with other downstream tasks using the multi-task training strategy discussed in later sections. A multi-layer fully connected network was applied to classification-type downstream tasks, including diagnostic tasks such as tumour type classification, primary site prediction and disease stage (i.e., primary tumour, recurrent tumour, metastatic tumour or normal control) prediction and demographic tasks, e.g., the prediction of gender. The output dimension of the classification downstream network was set to the number of classes. For the regression task, a similar network was attached to the deep embedding module, but only one neuron was kept in the output layer to predict the target scalar value (e.g., age of the subject). The survival prediction downstream network is more complicated and addressed separately in a subsequent section. The downstream networks add further regularisation to the low dimensional latent representation and urge the deep embedding module to learn the omics embedding related to certain downstream tasks. With the downstream modules, a single well-trained multi-task OmiEmbed network is able to reconstruct a comprehensive phenotype profile, including diagnostic, prognostic and demographic information from omics data.

### 3.4. Training Strategy

The same as the overall structure, the joint loss function is also comprised of two main components: the loss of the deep embedding and the loss of the downstream tasks.

We denote each type of input omics profile as $x_{j}$, and the corresponding reconstructed profile as $x_{j}^{\prime }$, where *j* is the omics type index and there are *M* omics types in total. The deep embedding loss can then be defined as follows:

(3) $$L_{embed}=\frac{1}{M}\sum_{j=1}^{M}BCE\left(x_{j},x_{j}^{\prime }\right)+D_{\mathrm{KL}}\left(N\left(\mu ,\sigma \right)∥N\left(0,I\right)\right)$$

where BCE is the binary cross-entropy to measure the distance between input data and reconstructed data, and the second term is the KL divergence between the learnt distribution and a standard Gaussian distribution.

In the downstream modules, each downstream task has its specific loss function $L_{down_{k}}$ and a corresponding weight $w_{k}$. For the classification type task, the loss function can be defined as:

(4) $$L_{classification}=CE\left(y,y^{\prime }\right)$$

where *y* is the ground truth, $y^{\prime }$ is the predicted label and CE is the cross-entropy loss. Similar to the classification loss, the loss function of regression type task is

(5) $$L_{regression}=MSE\left(y,y^{\prime }\right)$$

where MSE is the mean squared error between the real value and the predicted value. The loss function of the survival prediction task is discussed separately in the next section. The overall loss function of the downstream modules is the weighted sum of all downstream losses, i.e.,

(6) $$L_{down}=\frac{1}{K}\sum_{k=1}^{K}w_{k}L_{down_{k}}$$

where *K* is the number of downstream tasks, $L_{down_{k}}$ is the loss for a certain task and $w_{k}$ is the corresponding weight. $w_{k}$ can be manually set as hyperparameters or used as learnable parameters during the training process. In conclusion, the joint loss function of the end-to-end OmiEmbed network is

(7) $$L_{total}=\lambda L_{embed}+L_{down}$$

and depends on λ, which balances the two terms in the overall loss function.

Based on the aforementioned loss functions, three training phases were designed in OmiEmbed. Phase 1 was the unsupervised phase that only focused on the deep embedding module. In this training phase, only the deep embedding loss was backpropagated and only the parameters in the deep embedding network were updated based on the gradients. No label was required in the first training phase and this phase can be used separately as a dimensionality reduction or visualisation method. In Phase 2, the pre-trained embedding network was fixed whilst the downstream networks were being trained. The joint downstream loss was backpropagated and only the downstream networks were updated during this phase. After the embedding network and the downstream networks were pre-trained separately, the overall loss function defined in Equation (7) was calculated and backpropagated during Phase 3. In this final training phase the whole OmiEmbed network, including the deep embedding module and the downstream modules, was fine-tuned to obtain better performance.

### 3.5. Survival Prediction

Survival prediction is the most complicated downstream task implemented in OmiEmbed. The objective of this task is to obtain individual survival function and hazard function data for each subject, based on the high-dimensional omics data. The survival function can be denoted by

(8) S(t)=P[T>t]

where *T* is time elapsed between the sample collection time and the time of event occurring. The survival function signifies the probability that the failure event, i.e., death, has not occurred by time *t*. The hazard function can be defined as:

(9) $$h\left(t\right)=\lim_{dt\to 0}\frac{P[t\leq T<t+dt|T\geq t]}{dt}$$

which represents the instantaneous rate of occurrence for the failure event. A large hazard value manifests a great risk of death at specific time *t*. However, the original form of hazard function is infrequently used, and the risk score of each sample x is more commonly applied by subdividing the time axis into *m* time intervals, such that:

(10) $$r\left(x\right)=\sum_{i=1}^{m}h\left(t_{i},x\right).$$

In order to train a survival prediction downstream network, besides the omics data x, two elements of the survival labels are required: the event time *T* and the event indicator *E*. The indicator was set to 1 when the failure event was observed during the study and 0 when the event was not observed, which is termed censoring. In the case of censoring, the event time *T* is the time elapsed between the time when the sample was collected and the time of the last contact with the subject. Both *T* and *E* are available in the GDC dataset.

The multi-task logistic regression (MTLR) model [27] was applied and adapted to the OmiEmbed framework for the survival prediction downstream task. In the first step, the time axis was divided into *m* time intervals ${\left\{l_{i}\right\}}_{i=1}^{m}$. Each time interval was defined as $l_{i}=\left[t_{i-1},t_{i}\right)$, where $t_{0}$ = 0 and $t_{m}\geq max\left(T\right)$. The number of time intervals *m* is a hyperparameter. A larger *m* results in more fine-grained output, but requires more computation resources. We applied the multi-layer fully connected network as the backbone of our survival prediction network and the dimension of the output layer is the number of time intervals. As a result, the output of our survival prediction network is an *m*-dimensional vector $y^{\prime }=\left[y_{1}^{\prime },y_{2}^{\prime },\ldots ,y_{m}^{\prime }\right]$. Similarly, the survival label of each subject was also encoded into an *m*-dimensional vector $y=\left[y_{1},y_{2},\ldots ,y_{m}\right]$, where $y_{i}$ signifies the survival status of this subject at the time point $t_{i}$. The likelihood of observing y on the condition of sample x with the network parameters θ can be defined as follows:

(11) $$P_{\theta }\left(y|x\right)=\frac{\exp\left(\sum_{i=1}^{m}y_{i}y_{i}^{\prime }\right)}{\sum_{j=0}^{m}\exp\left(\sum_{i=j+1}^{m}y_{i}^{\prime }\right)}.$$

The objective of this survival network is to find a set of parameters θ that maximises the log-likelihood, hence the loss function of the survival prediction downstream task is defined as,

(12) $$L_{survival}=-\sum_{i=1}^{m}y_{i}y_{i}^{\prime }+\log\sum_{j=0}^{m}\exp\sum_{i=j+1}^{m}y_{i}^{\prime }$$

which can be directly applied to the downstream module and included in the joint loss function of OmiEmbed.

### 3.6. Multi-Task Strategy

With the joint loss function (6) of the multi-task downstream modules, we aimed to train multiple downstream networks in OmiEmbed simultaneously and efficiently instead of separate training, so as to obtain a unified model that can reconstruct a comprehensive phenotype profile for each subject. In order to balance the optimisation of different tasks, we adapted the multi-task optimisation method gradient normalisation (GradNorm) [28] to our OmiEmbed framework.

In Equation (6), $w_{k}$ is the weight of each downstream loss, and the weight can also be regarded as a trainable parameter that varies at each training iteration. The idea of GradNorm is to penalise the network if gradients of any downstream task are too large or too small, which makes all the tasks train at similar rates [28]. Firstly, the gradient norm of each downstream task is calculated by

(13) $$G_{\theta }^{\left(k\right)}={∥\nabla_{\theta }w_{k}L_{down_{k}}∥}_{2}$$

where θ is the parameters of the last encoding layer in the deep embedding module of OmiEmbed. The average gradient norm among all tasks can then be calculated by

(14) $${\bar{G}}_{\theta }=\frac{1}{K}\sum_{k=1}^{K}G_{\theta }^{\left(k\right)}$$

where *K* is the number of downstream tasks. The relative inverse training rate of each task can be defined as:

(15) $$r_{k}=\frac{{\tilde{L}}_{down_{k}}}{\frac{1}{K}\sum_{k=1}^{K}{\tilde{L}}_{down_{k}}}$$

where ${\tilde{L}}_{down_{k}}=L_{down_{k}}/L_{down_{k0}}$, which is the ratio of the current loss to the initial loss of the downstream task *k*. Then, the loss of GradNorm can be defined as:

(16) $$L_{grad}=\sum_{k=1}^{K}{\left|G_{\theta }^{\left(k\right)}-{\bar{G}}_{\theta }\times {r_{k}}^{\alpha }\right|}_{1}$$

where α is the hyperparameter that represents strength pulling tasks back to a common training rate. A separate backpropagation process was conducted during each training iteration on $L_{grad}$, which was only used for updating $w_{k}$.

## 4. Results

### 4.1. Implementation Details

The OmiEmbed multi-omics multi-task framework was built on the deep learning library PyTorch [29]. The code of OmiEmbed has been made open source on GitHub (https://github.com/zhangxiaoyu11/OmiEmbed/, accessed on 19 May 2021), and it is easy to apply it on any high-dimensional omics dataset for any aforementioned downstream task, individually or collaboratively. The detailed network structures of both the FC-type and CNN-type deep embedding modules were illustrated in Supplementary Figures S1 and S2. In the FC-type omics embedding network, CpG sites of DNA methylation profiles were separately connected to different hidden layers based on their targeting chromosomes in order to reduce the number of parameters, prevent overfitting and save the GPU memory. The chromosome separation step can be automatically processed in OmiEmbed with a built-in DNA methylation annotation if the FC-type embedding was selected using corresponding command-line arguments. Other deep learning techniques were also applied to prevent overfitting in OmiEmbed, including dropout [30], batch normalisation [31], weight decay regularisation and the learning rate schedule.

The model was trained on two NVIDIA Titan X GPUs with 12 gigabytes of memory each. The input dataset for each experiment was randomly separated into training, validation and testing sets. The separation was conducted in a stratified manner so as to keep the proportion of each class in all three sets. Stratified 5-fold cross-validation was also applied to robustly evaluate the performance of OmiEmbed and other compared methods avoiding bias from specific testing set. The open-source project files of OmiEmbed are well-organised with modular code structures, predefined packages and easy-to-follow tutorials, which make it convenient to extend the framework to other customised input data, network structures and downstream tasks.

### 4.2. Dimensionality Reduction

**Figure 2:**
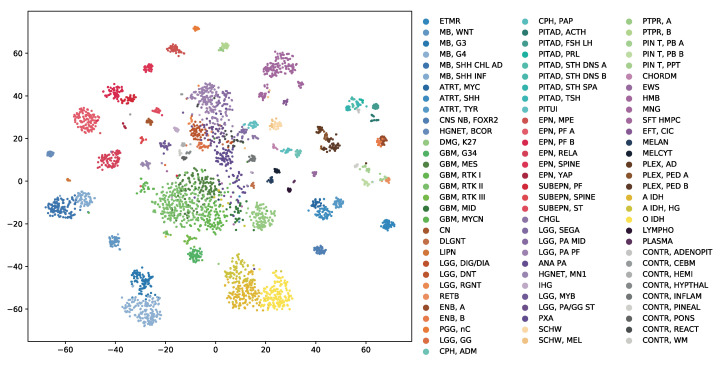
The 128D latent space of the BTM dataset learnt by the unsupervised phase of OmiEmbed. The scatter graph was visualised using t-SNE. Each label in the scatter graph was coloured by its methylation class label and the full name of each class abbreviation can be found in Supplementary Table S2. Tumour types belonging to the same upper-level class were marked in similar colours.

OmiEmbed can be regarded as an unsupervised dimensionality reduction method when only the training Phase 1 mentioned above was applied in the experiment. The high-dimensional multi-omics data can be compressed into a new representation with the target dimensionality set by the command line argument of OmiEmbed. Then, the output file can be directly used for visualisation or any other downstream task. Here, we reduced each sample in the BTM dataset into a 128D latent vector using the unsupervised Phase 1 of OmiEmbed. The learnt 128D latent space of the BTM dataset was visualised by t-distributed stochastic neighbour embedding (t-SNE) [32]. As illustrated in Figure 2, a multi-level hierarchical clustering pattern is revealed in the latent space. Each of the 82 brain tumour entities (e.g., chordoma, hemangioblastoma, and meningioma) and 9 normal control classes (e.g., normal chemispheric cortex, normal hypothalamus, and normal white matter) were automatically mapped into corresponding lower-level clusters. And tumour types belonging to the same upper-level class (e.g., glioblastoma, embryonal tumour, and ependymal tumour) also formed into the corresponding upper-level clusters with similar colours.

### 4.3. Tumour Classification

Instead of using the training Phase 1 individually as a dimensionality reduction method and separately training the downstream task with other machine learning algorithms, using all of the three training phases of OmiEmbed in an end-to-end manner is more efficient, with better performance. Here, we first tested the classification performance of OmiEmbed on the BTM dataset. There are two types of tumour type classification systems in this brain tumour dataset: the histopathological tumour type labels defined by the 2016 WHO classification [3] and the methylation tumour type labels defined by the original paper of this dataset [5]. For each type of these two classification systems, the 3905 samples were divided into more than 90 classes, including different normal control types (e.g., normal chemispheric cortex, normal hypothalamus, and normal white matter).

**Figure 3:**
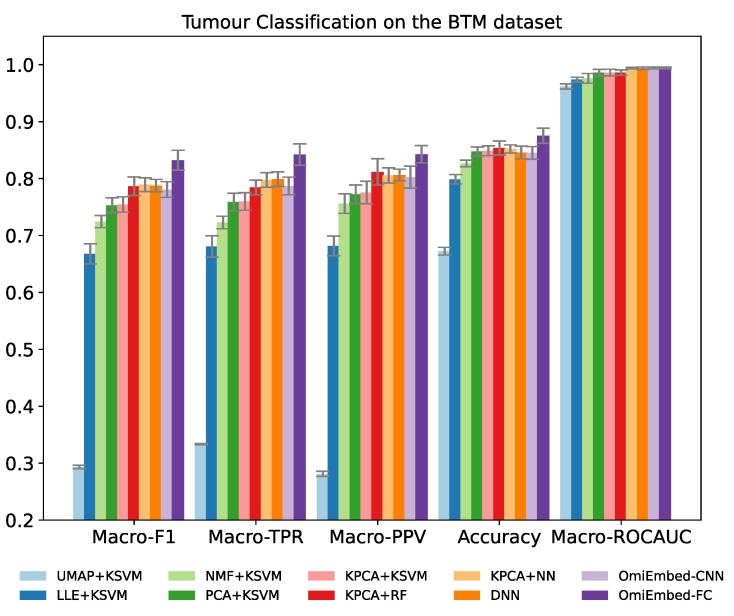
Performance comparison of OmiEmbed and other eight methods for the tumour entity classification task on the BTM dataset with the histopathological tumour type labels.

The classification performance was evaluated by five multi-class classification metrics: macro-averaged F1 score (Macro-F1), macro-averaged true positive rate (Macro-TPR), macro-averaged positive predictive value (Macro-PPV), overall accuracy and macro-averaged area under the receiver operating characteristic curve (Macro-ROCAUC). TPR is also known as sensitivity or recall and PPV is also known as precision. Both of the FC-type and CNN-type deep embedding modules of OmiEmbed were tested in the experiment. To adapt and exploit the local connectivity feature of convolutional layers, the order of the CpG sites in the input data was rearranged according to their targeting chromosome and location in that chromosome. Nevertheless, as shown in Table 2 and Figure 3, the performance of the CNN-type OmiEmbed was not as good as the performance of the FC-type OmiEmbed with statistical significance, which may be because omics data (e.g., gene expression and DNA methylation) do not meet the translation equivariance assumption of CNNs. Thus, the FC-type deep embedding module was selected for OmiEmbed in all of the following experiments.

The result of OmiEmbed was first compared with the combination of five dimensionality reduction methods and kernel support vector machine (KSVM). The five different dimensionality reduction methods included uniform manifold approximation and projection (UMAP) [33], locally linear embedding (LLE) [34], non-negative matrix factorization (NMF), principal component analysis (PCA) and kernel principal component analysis (KPCA) [35]. The original data from the BTM dataset were first reduced to 128D by the aforementioned dimensionality reduction methods and then classified by the KSVM with a radial basis function (RBF) kernel. The best results among them were achieved by KPCA, therefore, other machine learning methods were evaluated along with KPCA, including random forest (RF) [36] and neural network (NN). The NN used here was comprised of two hidden layers with 128 neurons and 64 neurons, respectively. The deep neural network (DNN) with the structure of 1024-512-256-128 was also compared with OmiEmbed in an end-to-end manner. As illustrated in Table 2, Figure 3, Supplementary Table S4 and Supplementary Figure S3, OmiEmbed achieved the best classification performance in all the five metrics, with both types of classification systems.

In stratified medicine, the confidence of certain diagnostic prediction is as important as the prediction itself. With the softmax layer in the classification downstream task module, OmiEmbed is able to output the predicted diagnosis, as well as the probability of every class for each input sample. Table 3 demonstrated three output examples from the testing set of the histopathological tumour type classification task on the BTM dataset. For testing samples like GSM2941340 and GSM2941792 which are difficult to determine the tumour type, OmiEmbed is able to not only predict the correct diagnosis, but detect analogous tumour entities (e.g., anaplastic astrocytoma IDH-mutant and diffuse astrocytoma IDH-mutant) and rank them by class probability.

### 4.4. Multi-Omics Integration

Different types of omics profiles can be integrated into single latent representation and used for different downstream tasks through the multi-omics deep embedding module of OmiEmbed. In order to test the effect of multi-omics integration on the downstream task, tumour type classifiers were trained on the GDC multi-omics dataset using OmiEmbed. Three types of omics data in the GDC dataset were used in the experiments: RNA-Seq gene expression, DNA methylation and miRNA expression. There are 33 tumour types and normal control class (34 classes in total) in the dataset. We trained the model with each omics type alone and two different multiple omics type combinations. The classification performance in each scenario was shown in Table 4. The performance metrics for each omics type alone were close to each other and the best metrics were achieved with the combination of all three omics types. This result indicates combining multiple omics data can yield better insights into the underlying mechanisms of diseases. It can also be observed that the classification performance using DNA methylation profile alone and miRNA expression profile alone was almost identical and the performance of the two different multi-omics combinations (i.e., gene expression+DNA mehyltaion and gene expression+DNA methylation+miRNA expression) was not significantly different, which indicated that miRNA expression profiles and DNA methylation profiles providing overlapping information for the tumour type classification task [37,38].

### 4.5. Reconstruction of Demographic and Clinical Features

With both the classification and regression downstream networks built in OmiEmbed, we were able to reconstruct a number of phenotype features from high-dimensional omics data. Here, we tested the prediction performance of four different phenotype features in the GDC dataset, including age, gender, the disease stage and primary site of the clinical sample. Detailed information of each categorical features was listed in Supplementary Table S5.

The disease stage is the clinical type of the sample, which consists of primary tumour, metastatic tumour, recurrent tumour and normal control tissue. The primary site is the place where the cancer starts growing. Samples in the GDC dataset are from 28 different primary sites such as breast, kidney, lung, and skin. As for the gender of each sample, since the molecular features targeting the Y chromosome were filtered in the preprocessing stage, the model was required to classify the gender based on other molecular features. The OmiEmbed classification performance of the three categorical phenotype features was shown in Table 5, along with the results of a DNN with the structure of 1024-512-256-128. The full comparison of all nine methods was illustrated in Supplementary Tables S6–S8 and Supplementary Figures S4–S6.

**Figure 4:**
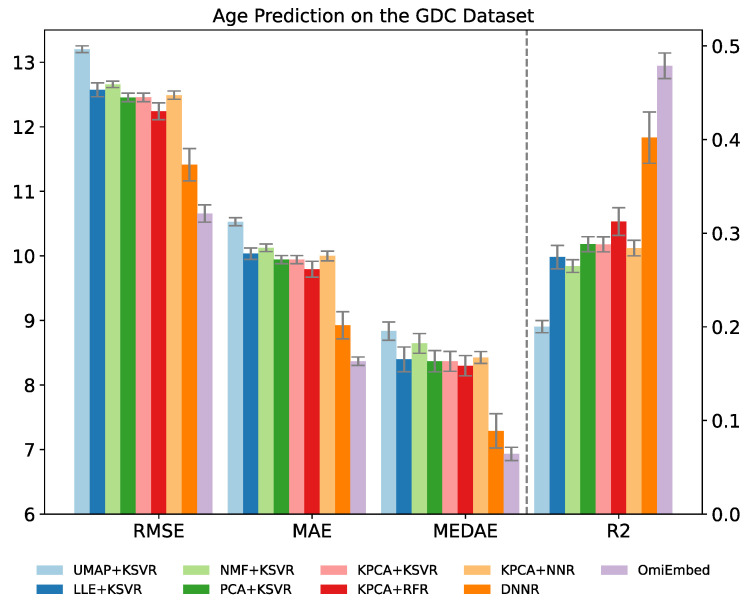
Performance comparison of OmiEmbed and other eight methods for the age prediction task on the GDC dataset. For root mean square error (RMSE), mean absolute error (MAE) and median absolute error (MEDAE), lower values mean better regression performance. For $R^{2}$ score, higher values mean better regression performance.

Since the label of age is numerical instead of categorical, the regression downstream module was applied for the age prediction task. The performance of age prediction was evaluated by the three regression metrics: median absolute error, mean absolute error, root mean square error (RMSE) and coefficient of determination ($R^{2}$), which was illustrated in Table 6 and Figure 4. For median absolute error, mean absolute error and RMSE lower values represent better regression performance, whereas for $R^{2}$ score higher values indicate better regression performance.

The age prediction performance of OmiEmbed was first compared with the combination of the five aforementioned dimensionality reduction methods and kernel support vector regressor (KSVR). The original data from the GDC dataset were first reduced to 128D and then fed to the KSVR with the RBF kernel. Other regression methods were also evaluated along with KPCA, including random forest regressor (RFR) [36] and neural network regressor (NNR). The NNR adopted here was comprised of two hidden layers with 128 neurons and 64 neurons, respectively. The deep neural network regressor (DNNR) with the structure of 1024-512-256-128 was also compared with OmiEmbed in an end-to-end manner. OmiEmbed achieved the best regression performance with the lowest distance error and highest coefficient of determination.

### 4.6. Survival Prediction

**Figure 5:**
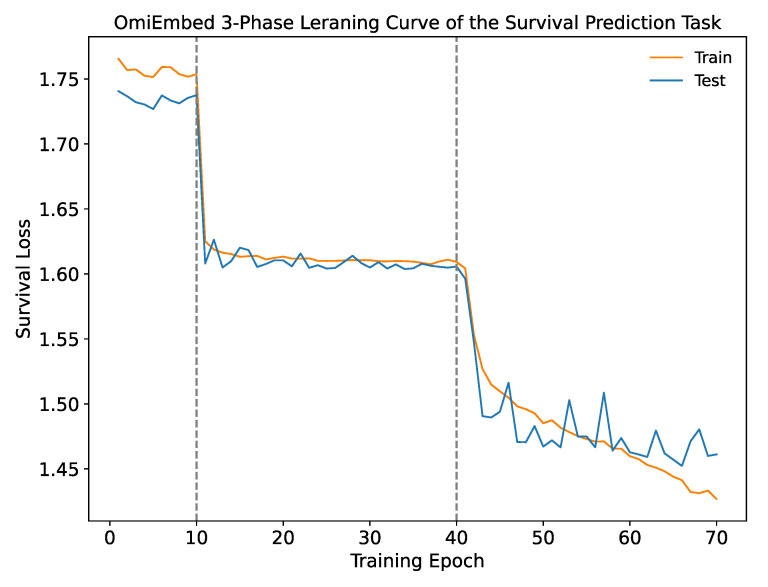
The learning curve of the survival prediction task with the three-phase training strategy of OmiEmbed. Epoch 1 to 10 belong to Phase 1; epoch 11 to 40 belong to Phase 2; epoch 41 to 70 belong to Phase 3.

With the survival prediction downstream module of OmiEmbed, we are able to predict the survival function of each subject from corresponding high-dimensional omics data. Just like other downstream tasks, OmiEmbed was trained by the three-phase training strategy for the survival prediction task. Survival losses at each epoch on the training and testing set were illustrated in Figure 5 with a step shape learning curve. The first ten epochs were in Phase 1, where the embedding network was pretrained in an unsupervised manner. In epoch 11 to epoch 40, the downstream network was trained individually when the pre-trained embedding network was fixed, and in the last phase, the whole network was fine-tuned for better performance, which is consistent with the learning curve.

The performance of the survival prediction downstream task was evaluated by concordance index (C-index) and integrated Brier score (IBS), which are the most commonly used metrics for survival prediction. A C-index value of 1 indicates the perfect prediction model and a value of 0.5 signifies that the performance of the model is similar to expected at random. The Brier score indicates the accuracy of a predicted survival function at a certain time point, which is between 0 and 1. IBS is the average Brier score among all available times, providing an overall calculation of the model performance.

**Figure 6:**
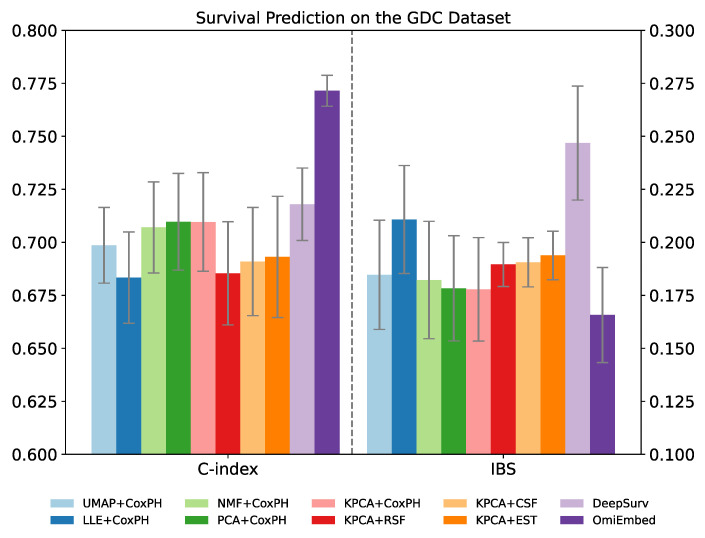
Performance comparison of OmiEmbed and other nine methods for the survival prediction task on the GDC dataset. For C-index, higher values mean better prediction performance. For IBS score, lower values mean better prediction performance.

The results of OmiEmbed were compared with methods that first reduced the dimensionality of input omics data to 128D using UMAP [33], LLE [34], NMF, PCA or KPCA [35], and then fed the 128D latent vectors to the survival prediction method Cox proportional hazard model (CoxPH). Other survival prediction methods, including random survival forest (RSF) [39], conditional survival forest (CSF) [40] and extra survival trees (EST) [41], were also evaluated after being reduced to 128D latent vectors by KPCA. The survival prediction performance of OmiEmbed was also compared with the state-of-the-art deep learning method DeepSurv [42]. OmiEmbed got the best C-index (0.7715) and IBS (0.1657) among all of the ten methods, as shown in Table 7 and Figure 6.

**Figure 7:**
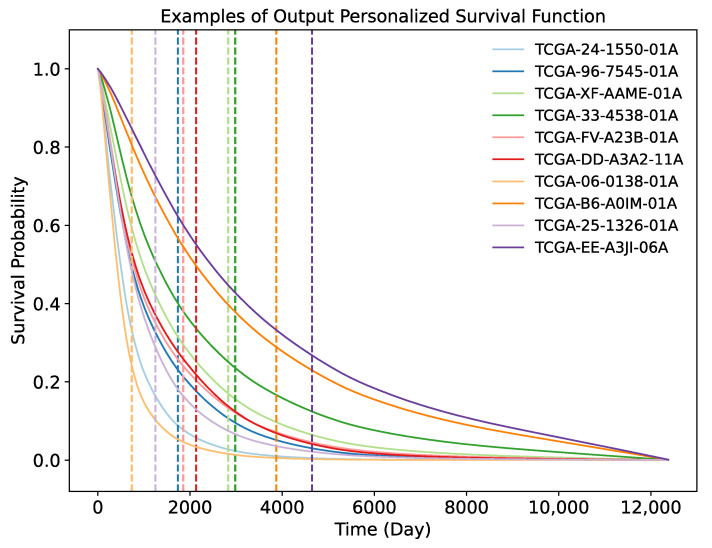
Personalised survival curves of ten random subjects from the testing set of the GDC dataset. The dashed vertical line with the corresponding colour indicates the death time of each subject.

OmiEmbed is able to output the personalised survival function based on the corresponding omics profile. As illustrated in Figure 7, we randomly selected ten subjects with their observed death time from the testing set of the GDC dataset as examples and plotted the survival curve for each of them. The actual death time of each subject was also marked in the figure by the dashed vertical line with the corresponding colour.

### 4.7. Multi-Task Learning

Instead of training each aforementioned downstream module separately, we can also train multiple downstream modules together using the multi-task training strategy expatiated in the previous section. With the multi-task strategy, OmiEmbed is able to perform diverse downstream tasks simultaneously and reconstruct a comprehensive phenotype profile of each subject from high-dimensional omics data, using one unified network in one forward propagation. In order to test the multi-task performance of OmiEmbed, we first selected three typical downstream tasks belonging to three distinct categories, the survival prediction task, the tumour type classification task and the age regression task, for the evaluation. Three downstream modules along with the deep embedding module were trained collaboratively using the joint loss function Equation (7) and the GradNorm loss function Equation (16). As shown in Table 8, the performance is higher in all three downstream tasks when they were trained in a unified multi-task OmiEmbed network, compared with being trained separately.

**Figure 8:**
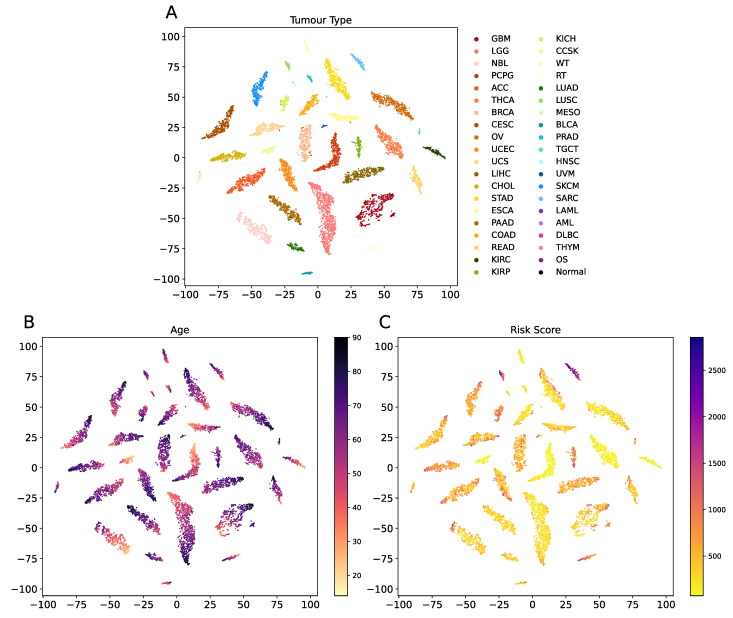
Visualisation of the latent space learnt by the multi-task OmiEmbed. Each sample was colour by its tumour type (**A**), age (**B**) and risk score (**C**) in three corresponding scatter graphs with the same latent representation. Apparent patterns related to the three types of labels can be seen in the scatter graphs.

Since different downstream networks shared the common deep embedding module with the multi-task learning strategy, the latent representation learnt by multi-task OmiEmbed contained comprehensive information of each downstream task. The learnt omics embedding reduced the dimensionality of each sample in the GDC dataset, which was then visualised using t-SNE and illustrated in Figure 8. Each sample was coloured by its tumour type, age and risk score in three corresponding scatter graphs with the same latent representation, which revealed apparent patterns related to the three types of labels.

## 5. Conclusions

OmiEmbed is an open-source deep learning framework designed for multi-omics data analysis, with tasks including dimensionality reduction, multi-omics data integration, tumour type classification, disease stage prediction, demographic label reconstruction and prognosis prediction. All of the aforementioned tasks can be performed individually or collaboratively by a unified architecture, which is comprised of the deep embedding and downstream task modules. OmiEmbed achieved promising results in each downstream task outperforming state-of-the-art methods, and obtained a better performance with the multi-task strategy comparing to training them individually. The multi-task OmiEmbed learnt a single embedding for all of the downstream tasks, which contained comprehensive information in the latent space. Our results indicated that OmiEmbed was able to reconstruct a comprehensive profile of each subject, including demographic, diagnostic and prognostic information from the multi-omics data, which has a great potential to facilitate more accurate and personalised clinical decision making. OmiEmbed is publicly available with modular code structures, predefined packages and easy-to-follow tutorials, which make the unified framework applicable to any omics type and downstream task with minimal modification. We believe that OmiEmbed will also become a framework for other researchers to analyse high-dimensional omics data using the deep learning and multi-task learning methodology.

In the future work, we plan to apply a Gaussian mixture model (GMM) [43,44] as the prior distribution of the latent variables in the deep embedding module. With the biological network data including protein–protein interactions (PPI) networks [45], gene regulatory networks (GRN) [46,47], gene co-expression networks [48], etc., we are going to apply the emerging graph neural network (GNN) technologies [49] to improve the performance of multi-omics integration. How to deal with biases in the model is a crucial issue for medical applications of machine learning. We plan to adopt the latest intra-processing method [50] to debias neural networks for a more trustworthy OmiEmbed. Model interpretability is another potential improvement of the OmiEmbed framework. XOmiVAE [51] has taken the step to implement and analyse the interpretability of multi-omics deep learning models, which will be integrated to OmiEmbed in our future work to fully open the “black box”.

## Figures and Tables

**Table 1:** Overview information of the Genomic Data Commons (GDC) pan-cancer dataset and the GSE109381 brain tumour methylation (BTM) dataset. The GDC dataset includes two cancer genome programmes: The Cancer Genome Atlas (TCGA) and Therapeutically Applicable Research to Generate Effective Treatment (TARGET).

Dataset Info	GDC			BTM
Domain	Pan-cancer			Brain tumour
Tumour type	33 (TCGA) + 3 (TARGET) + 1 (normal)			86 + 8 (normal)
Additional label	Disease stage, primary site, gender, age, survival			Disease stage, gender, age
Omics type	Gene expression	DNA methylation	miRNA expression	DNA methylation
Feature number	60,483	485,577	1881	485,577
Sample number	11,538	9736	11,020	3905

**Table 2:** The classification performance on the BTM dataset using the histopathological tumour type labels (2016 WHO classification) with 5-fold cross-validation, which was measured by macro-averaged F1 score (Macro-F1), macro-averaged true positive rate (Macro-TPR), macro-averaged positive predictive value (Macro-PPV), overall accuracy and macro-averaged area under the receiver operating characteristic curve (Macro-ROCAUC).

Method	Macro-F1	Macro-TPR	Macro-PPV	Accuracy	Macro-ROCAUC
UMAP + KSVM	0.2933 ± 0.0029	0.3334 ± 0.0012	0.2812 ± 0.0044	0.6725 ± 0.0067	0.9619 ± 0.0045
LLE + KSVM	0.6678 ± 0.0178	0.6807 ± 0.0189	0.6817 ± 0.0172	0.7987 ± 0.0089	0.9740 ± 0.0041
NMF + KSVM	0.7245 ± 0.0107	0.7228 ± 0.0110	0.7560 ± 0.0172	0.8266 ± 0.0057	0.9761 ± 0.0085
PCA + KSVM	0.7531 ± 0.0131	0.7588 ± 0.0155	0.7724 ± 0.0163	0.8477 ± 0.0078	0.9861 ± 0.0058
KPCA + KSVM	0.7544 ± 0.0135	0.7598 ± 0.0153	0.7755 ± 0.0199	0.8488 ± 0.0087	0.9863 ± 0.0058
KPCA + RF	0.7865 ± 0.0162	0.7844 ± 0.0129	0.8117 ± 0.0233	0.8537 ± 0.0122	0.9862 ± 0.0046
KPCA + NN	0.7893 ± 0.0120	0.7975 ± 0.0127	0.8056 ± 0.0134	0.8521 ± 0.0070	0.9940 ± 0.0015
DNN	0.7877 ± 0.0108	0.7991 ± 0.0128	0.8063 ± 0.0103	0.8459 ± 0.0112	0.9940 ± 0.0021
OmiEmbed-CNN	0.7807 ± 0.0138	0.7869 ± 0.0156	0.8026 ± 0.0193	0.8452 ± 0.0111	0.9943 ± 0.0017
**OmiEmbed-FC**	**0.8323 ± 0.0174**	**0.8421 ± 0.0188**	**0.8429 ± 0.0152**	**0.8754 ± 0.0133**	**0.9943 ± 0.0016**

The best results were marked in bold.

**Table 3:** Three output examples from the testing set of histopathological tumour type classification on the BTM dataset. Ground truth (GT) is the pathological diagnosis made by clinicians.

Sample ID	Rank	Class ID	Class Name (2016 WHO Classification of CNS Tumours)	Probability
GSM2941340	1st	4	Anaplastic ependymoma	75.3695%
	2nd	5	Ependymoma	21.5774%
	3rd	23	Myxopapillary ependymoma	0.0275%
	4th	62	Anaplastic (malignant) meningioma	0.0008%
	5th	31	Atypical meningioma	0.0004%
	**GT**	**4**	**Anaplastic ependymoma**	
GSM2941792	1st	6	Anaplastic astrocytoma, IDH-mutant	81.4425%
	2nd	13	Diffuse astrocytoma, IDH-mutant	17.8859%
	3rd	15	Oligodendroglioma, IDH-mutant and 1p/19q-codeleted	0.4496%
	4th	10	Anaplastic oligodendroglioma, IDH-mutant and 1p/19q-codeleted	0.1553%
	5th	54	Anaplastic astrocytoma, IDH-wildtype	0.0124%
	**GT**	**6**	**Anaplastic astrocytoma, IDH-mutant**	
GSM2405444	1st	0	Glioblastoma, IDH-wildtype	99.9995%
	2nd	32	Gliosarcoma, IDH-wildtype	0.0001%
	3rd	44	Anaplastic pilocytic astrocytoma	0.0001%
	4th	52	Anaplastic pilocytic astrocytoma (unresolved status)	0.0001%
	5th	20	Ganglioglioma	0.0001%
	**GT**	**0**	**Glioblastoma, IDH-wildtype**	

The ground truth diagnoses were marked in bold.

**Table 4:** The performance of tumour type classification on the GDC multi-omics dataset with different omics type combinations.

Omics Type	Macro-F1	Macro-TPR	Macro-PPV	Accuracy	Macro-ROCAUC
Gene expression (a)	0.9518 ± 0.0053	0.9522 ± 0.0055	0.9558 ± 0.0069	0.9676 ± 0.0027	0.9982 ± 0.0003
DNA methylation (b)	0.9273 ± 0.0167	0.9253 ± 0.0192	0.9333 ± 0.0181	0.9650 ± 0.0040	0.9985 ± 0.0001
miRNA expression (c)	0.9274 ± 0.0140	0.9268 ± 0.0134	0.9320 ± 0.0148	0.9544 ± 0.0057	0.9983 ± 0.0004
Multi-omics (a + b)	0.9675 ± 0.0083	0.9669 ± 0.0077	0.9643 ± 0.0077	0.9753 ± 0.0040	0.9988 ± 0.0009
**Multi-omics (a + b + c)**	**0.9683 ± 0.0020**	**0.9684 ± 0.0026**	**0.9705 ± 0.0047**	**0.9771 ± 0.0027**	**0.9991 ± 0.0002**

The best results were marked in bold.

**Table 5:** The classification performance of predicting three categorical phenotype features on the GDC dataset.

Label	Method	Macro-F1	Macro-TPR	Macro-PPV	Accuracy	Macro-ROCAUC
Disease stage	DNN	0.7530 ± 0.0035	0.7552 ± 0.0090	0.7517 ± 0.0105	0.9782 ± 0.0014	0.9552 ± 0.0207
	OmiEmbed	0.8173 ± 0.0401	0.8016 ± 0.0291	0.8610 ± 0.0816	0.9797 ± 0.0024	0.9540 ± 0.0320
Primary site	DNN	0.9639 ± 0.0066	0.9638 ± 0.011	0.9576 ± 0.0123	0.9593 ± 0.0116	0.9987 ± 0.0006
	OmiEmbed	0.9717 ± 0.0066	0.9711 ± 0.0046	0.9734 ± 0.0095	0.9812 ± 0.0023	0.9994 ± 0.0003
Gender	DNN	0.8701 ± 0.0725	0.887 ± 0.0413	0.8713 ± 0.0705	0.8668 ± 0.079	0.962 ± 0.0102
	OmiEmbed	0.9560 ± 0.0023	0.9558 ± 0.0024	0.9568 ± 0.0018	0.9561 ± 0.0022	0.9903 ± 0.0019

**Table 6:** The age prediction performance of OmiEmbed and eight other methods on the GDC dataset. For median absolute error, mean absolute error and RMSE, lower values mean better regression performance. For $R^{2}$ score, higher values mean better regression performance.

Method	Median Absolute Error	Mean Absolute Error	RMSE	$R^{2}$
UMAP + KSVR	8.8353 ± 0.1408	10.5292 ± 0.0621	13.2020 ± 0.0519	0.2003 ± 0.0065
LLE + KSVR	8.3977 ± 0.1910	10.0348 ± 0.0885	12.5742 ± 0.1075	0.2745 ± 0.0125
NMF + KSVR	8.6448 ± 0.1534	10.1268 ± 0.0589	12.6571 ± 0.0498	0.2649 ± 0.0069
PCA + KSVR	8.3691 ± 0.1646	9.9430 ± 0.0633	12.4547 ± 0.0669	0.2882 ± 0.0080
KPCA + KSVR	8.3674 ± 0.1544	9.9432 ± 0.0633	12.4561 ± 0.0666	0.2881 ± 0.0080
KPCA + RFR	8.2990 ± 0.1576	9.7939 ± 0.1225	12.2403 ± 0.1307	0.3125 ± 0.0148
KPCA + NNR	8.4269 ± 0.0917	9.9995 ± 0.0752	12.4907 ± 0.0649	0.2841 ± 0.0083
DNNR	7.2897 ± 0.2649	8.9247 ± 0.2101	11.4128 ± 0.2490	0.4020 ± 0.0273
**OmiEmbed**	**6.9330 ± 0.1031**	**8.3694 ± 0.0648**	**10.6578 ± 0.1337**	**0.4788 ± 0.0137**

The best results were marked in bold.

**Table 7:** The survival prediction performance of OmiEmbed and nine other methods on the GDC dataset. For C-index, higher values mean better prediction performance. For IBS score, lower values mean better prediction performance.

Mathod	C-Index	IBS
UMAP + CoxPH	0.6986 ± 0.0179	0.1847 ± 0.0258
LLE + CoxPH	0.6833 ± 0.0215	0.2108 ± 0.0255
NMF + CoxPH	0.7070 ± 0.0215	0.1822 ± 0.0277
PCA + CoxPH	0.7097 ± 0.0228	0.1783 ± 0.0248
KPCA + CoxPH	0.7096 ± 0.0233	0.1778 ± 0.0244
KPCA + RSF	0.6854 ± 0.0244	0.1896 ± 0.0104
KPCA + CSF	0.6909 ± 0.0255	0.1906 ± 0.0116
KPCA + EST	0.6931 ± 0.0286	0.1938 ± 0.0115
DeepSurv	0.7180 ± 0.0171	0.2468 ± 0.0269
**OmiEmbed**	**0.7715 ± 0.0073**	**0.1657 ± 0.0224**

The best results were marked in bold.

**Table 8:** Multi-task training performance of OmiEmbed with three typical downstream tasks. For C-index, macro-F1 and accuracy, higher values mean better prediction performance. For IBS score, RMSE and median absolute error, lower values mean better prediction performance.

	Survival		Tumour Type		Age	
	C-Index	IBS	Macro-F1	Accuracy	RMSE	Median Absolute Error
Single task alone	0.7715 ± 0.0073	0.1657 ± 0.0224	0.9518 ± 0.0053	0.9676 ± 0.0027	10.6578 ± 0.1337	6.9330 ± 0.1031
Multi-task	0.7823 ± 0.0076	0.1590 ± 0.0212	0.9653 ± 0.0057	0.9733 ± 0.0029	10.6336 ± 0.1034	6.6759 ± 0.1195

## Data Availability

The source code, user guide document, and built-in annotation file have been made publicly available on GitHub (https://github.com/zhangxiaoyu11/OmiEmbed/, accessed on 19 May 2021). The release of OmiEmbed has also been stored in Zenodo under the doi:10.5281/zenodo.4854112. The BTM dataset is available from GEO (https://www.ncbi.nlm.nih.gov/geo/query/acc.cgi?acc=GSE109381, accessed on 30 August 2019) with the accession ID GSE109381. The harmonised GDC pan-cancer dataset can be downloaded from the UCSC Xena data portal (https://xenabrowser.net/datapages/, accessed on 1 May 2019).

## Supplementary Materials

The following are available online at https://www.mdpi.com/article/10.3390/cancers13123047/s1, Figure S1: The detailed network structure for the CNN-type deep embedding module in OmiEmbed, Figure S2: The detailed network structure for the FC-type deep embedding module in OmiEmbed, Figure S3: Performance comparison of OmiEmbed and other eight methods for the tumour entity classification task on the BTM dataset with the methylation tumour type labels, Figure S4: Performance comparison of OmiEmbed and other eight methods for the disease stage classification task on the GDC dataset, Figure S5: Performance comparison of OmiEmbed and other eight methods for the primary site classification task on the GDC dataset, Figure S6: Performance comparison of OmiEmbed and other eight methods for the gender classification task on the GDC dataset, Table S1: Tumour type information of the GDC pan-cancer dataset, Table S2: Detailed tumour type information of the BTM dataset with the methylation class labels, Table S3: Detailed tumour type information of the BTM dataset with the pathological class labels defined by the 2016 WHO classification of CNS tumours, Table S4: The classification performance on the BTM dataset using the methylation tumour type labels with 5-fold cross-validation, Table S5: Detailed information for the categorical features predicted by OmiEmbed on the GDC dataset, Table S6: The disease stage classification performance of OmiEmbed and eight other methods on the GDC dataset, Table S7: The primary site classification performance of OmiEmbed and eight other methods on the GDC dataset, Table S8: The gender classification performance of OmiEmbed and eight other methods on the GDC dataset.

## References

[1] Hasin Y., Seldin M.M., Lusis A. (2017). Multi-omics approaches to disease. Genome Biol..

[2] Berger B., Peng J., Singh M. (2013). Computational solutions for omics data. Nat. Rev. Genet..

[3] Louis D.N., Perry A., Reifenberger G., Von Deimling A., Figarella-Branger D., Cavenee W.K., Ohgaki H., Wiestler O.D., Kleihues P., Ellison D.W. (2016). The 2016 World Health Organization classification of tumors of the central nervous system: A summary. Acta Neuropathol..

[4] Chaudhary K., Poirion O.B., Lu L., Garmire L. (2017). Deep Learning–Based Multi-Omics Integration Robustly Predicts Survival in Liver Cancer. Clin. Cancer Res..

[5] Capper D., Jones D.T., Sill M., Hovestadt V., Schrimpf D., Sturm D., Koelsche C., Sahm F., Chavez L., Reuss D.E. (2018). DNA methylation-based classification of central nervous system tumours. Nature.

[6] Zhang X., Zhang J., Sun K., Yang X., Dai C., Guo Y. Integrated Multi-omics Analysis Using Variational Autoencoders: Application to Pan-cancer Classification. Proceedings of the 2019 IEEE International Conference on Bioinformatics and Biomedicine (BIBM).

[7] Amodio M., van Dijk D., Srinivasan K., Chen W., Mohsen H., Moon K.R., Campbell A., Zhao Y., Wang X., Venkataswamy M. (2019). Exploring single-cell data with deep multitasking neural networks. Nat. Methods.

[8] Goodfellow I., Bengio Y., Courville A. (2016). Deep Learning.

[9] Voulodimos A., Doulamis N., Doulamis A., Protopapadakis E. (2018). Deep Learning for Computer Vision: A Brief Review. Comput. Intell. Neurosci..

[10] Young T., Hazarika D., Poria S., Cambria E. (2018). Recent Trends in Deep Learning Based Natural Language Processing. IEEE Comput. Intell. Mag..

[11] Ding J., Condon A., Shah S. (2018). Interpretable dimensionality reduction of single cell transcriptome data with deep generative models. Nat. Commun..

[12] Lopez R., Regier J., Cole M., Jordan M.I., Yosef N. (2018). Deep Generative Modeling for Single-cell Transcriptomics. Nat. Methods.

[13] Eraslan G., Simon L., Mircea M., Mueller N., Theis F. (2019). Single-cell RNA-seq denoising using a deep count autoencoder. Nat. Commun..

[14] Way G.P., Greene C. (2018). Extracting a biologically relevant latent space from cancer transcriptomes with variational autoencoders. Pac. Symp. Biocomput. Pac. Symp. Biocomput..

[15] Ma T., Zhang A. Affinitynet: Semi-supervised few-shot learning for disease type prediction. Proceedings of the AAAI Conference on Artificial Intelligence.

[16] Cheerla A., Gevaert O. (2019). Deep learning with multimodal representation for pancancer prognosis prediction. Bioinformatics.

[17] Grossman R.L., Heath A.P., Ferretti V., Varmus H.E., Lowy D.R., Kibbe W.A., Staudt L.M. (2016). Toward a shared vision for cancer genomic data. N. Engl. J. Med..

[18] Danaee P., Ghaeini R., Hendrix D. (2017). A Deep Learning Approach for Cancer Detection and Relevant Gene Identification. Pac. Symp. Biocomput. Pac. Symp. Biocomput..

[19] Lyu B., Haque A. Deep Learning Based Tumor Type Classification Using Gene Expression Data. Proceedings of the 2018 ACM International Conference on Bioinformatics, Computational Biology, and Health Informatics.

[20] Rhee S., Seo S., Kim S. (2018). Hybrid Approach of Relation Network and Localized Graph Convolutional Filtering for Breast Cancer Subtype Classification. arXiv.

[21] Jurmeister P., Bockmayr M., Seegerer P., Bockmayr T., Treue D., Montavon G., Vollbrecht C., Arnold A., Teichmann D., Bressem K. (2019). Machine learning analysis of DNA methylation profiles distinguishes primary lung squamous cell carcinomas from head and neck metastases. Sci. Transl. Med..

[22] Huang Z., Zhan X., Xiang S., Johnson T., Helm B., Yu C.Y., Zhang J., Salama P., Rizkalla M., Han Z. (2019). SALMON: Survival Analysis Learning With Multi-Omics Neural Networks on Breast Cancer. Front. Genet..

[23] Azarkhalili B., Saberi A., Chitsaz H., Sharifi-Zarchi A. (2019). DeePathology: Deep Multi-Task Learning for Inferring Molecular Pathology from Cancer Transcriptome. Sci. Rep..

[24] Weinstein J.N., Collisson E.A., Mills G.B., Shaw K.R.M., Ozenberger B.A., Ellrott K., Shmulevich I., Sander C., Stuart J.M., Network C.G.A.R. (2013). The cancer genome atlas pan-cancer analysis project. Nat. Genet..

[25] Aryee M.J., Jaffe A.E., Corrada-Bravo H., Ladd-Acosta C., Feinberg A.P., Hansen K.D., Irizarry R.A. (2014). Minfi: A flexible and comprehensive Bioconductor package for the analysis of Infinium DNA methylation microarrays. Bioinformatics.

[26] Kingma D.P., Welling M. (2013). Auto-encoding variational bayes. arXiv.

[27] Yu C.N., Greiner R., Lin H.C., Baracos V. (2011). Learning patient-specific cancer survival distributions as a sequence of dependent regressors. Adv. Neural Inf. Process. Syst..

[28] Chen Z., Badrinarayanan V., Lee C.Y., Rabinovich A. Gradnorm: Gradient normalization for adaptive loss balancing in deep multitask networks. Proceedings of the International Conference on Machine Learning.

[29] Paszke A., Gross S., Massa F., Lerer A., Bradbury J., Chanan G., Killeen T., Lin Z., Gimelshein N., Antiga L. (2019). PyTorch: An Imperative Style, High-Performance Deep Learning Library. arXiv.

[30] Srivastava N., Hinton G., Krizhevsky A., Sutskever I., Salakhutdinov R. (2014). Dropout: A simple way to prevent neural networks from overfitting. J. Mach. Learn. Res..

[31] Ioffe S., Szegedy C. Batch normalization: Accelerating deep network training by reducing internal covariate shift. Proceedings of the International Conference on Machine Learning.

[32] Van der Maaten L., Hinton G. (2008). Visualizing data using t-SNE. J. Mach. Learn. Res..

[33] McInnes L., Healy J., Melville J. (2018). Umap: Uniform manifold approximation and projection for dimension reduction. arXiv.

[34] Roweis S., Saul L. (2000). Nonlinear dimensionality reduction by locally linear embedding. Science.

[35] Schölkopf B., Smola A., Müller K.R. (1997). Kernel principal component analysis. International Conference on Artificial Neural Networks.

[36] Breiman L. (2004). Random Forests. Mach. Learn..

[37] Aure M.R., Fleischer T., Bjørklund S., Ankill J., Castro-Mondragón J., Børresen-Dale A., Tost J., Sahlberg K., Mathelier A., Tekpli X. (2021). Crosstalk between microRNA expression and DNA methylation drives the hormone-dependent phenotype of breast cancer. Genome Med..

[38] Wang S., Wu W., Claret F. (2017). Mutual regulation of microRNAs and DNA methylation in human cancers. Epigenetics.

[39] Ishwaran H., Kogalur U.B., Blackstone E., Lauer M. (2008). Random survival forests. Ann. Appl. Stat..

[40] Wright M.N., Dankowski T., Ziegler A. (2017). Unbiased split variable selection for random survival forests using maximally selected rank statistics. Stat. Med..

[41] Geurts P., Ernst D., Wehenkel L. (2006). Extremely randomized trees. Mach. Learn..

[42] Katzman J., Shaham U., Cloninger A., Bates J., Jiang T., Kluger Y. (2018). DeepSurv: Personalized treatment recommender system using a Cox proportional hazards deep neural network. BMC Med. Res. Methodol..

[43] Zong B., Song Q., Min M.R., Cheng W., Lumezanu C., ki Cho D., Chen H. Deep Autoencoding Gaussian Mixture Model for Unsupervised Anomaly Detection. Proceedings of the International Conference on Learning Representations.

[44] Zhu Y., Tang Y., Tang Y., Elton D., Lee S., Pickhardt P., Summers R. (2020). Cross-Domain Medical Image Translation by Shared Latent Gaussian Mixture Model. arXiv.

[45] Szklarczyk D., Franceschini A., Wyder S., Forslund K., Heller D., Huerta-Cepas J., Simonovic M., Roth A.C.J., Santos A., Tsafou K. (2015). STRING v10: Protein–protein interaction networks, integrated over the tree of life. Nucleic Acids Res..

[46] Ogata H., Goto S., Sato K., Fujibuchi W., Bono H., Kanehisa M. (1999). KEGG: Kyoto Encyclopedia of Genes and Genomes. Nucleic Acids Res..

[47] Fabregat A., Jupe S., Matthews L., Sidiropoulos K., Gillespie M., Garapati P., Haw R., Jassal B., Korninger F., May B. (2018). The Reactome pathway knowledgebase. Nucleic Acids Res..

[48] Obayashi T., Hayashi S., Shibaoka M., Saeki M., Ohta H., Kinoshita K. (2008). COXPRESdb: A database of coexpressed gene networks in mammals. Nucleic Acids Res..

[49] Wu Z., Pan S., Chen F., Long G., Zhang C., Yu P.S. (2021). A Comprehensive Survey on Graph Neural Networks. IEEE Trans. Neural Networks Learn. Syst..

[50] Savani Y., White C., Govindarajulu N.S. (2020). Intra-Processing Methods for Debiasing Neural Networks. Adv. Neural Inf. Process. Syst..

[51] Withnell E., Zhang X., Sun K., Guo Y. (2021). XOmiVAE: An interpretable deep learning model for cancer classification using high-dimensional omics data. arXiv.

