# Supplementary material for: OmiEmbed: A Unified Multi-Task Deep Learning Framework for Multi-Omics Data
Source: Cancers (Basel). 2021 Jun 18;13(12):3047. doi: 10.3390/cancers13123047 (PMC8235477; doi:10.3390/cancers13123047)
Supplement: Supplementary file 1 [file cancers-13-03047-s001.zip › cancers-1248175-supplementary.pdf]

OmiEmbed: a unified multi-task deep learning framework for multi-omics data – Supplementary information

Xiaoyu Zhang, Yuting Xing, Kai Sun, Yike Guo

This document provides the Supplementary Tables and Figures mentioned in the manuscript.

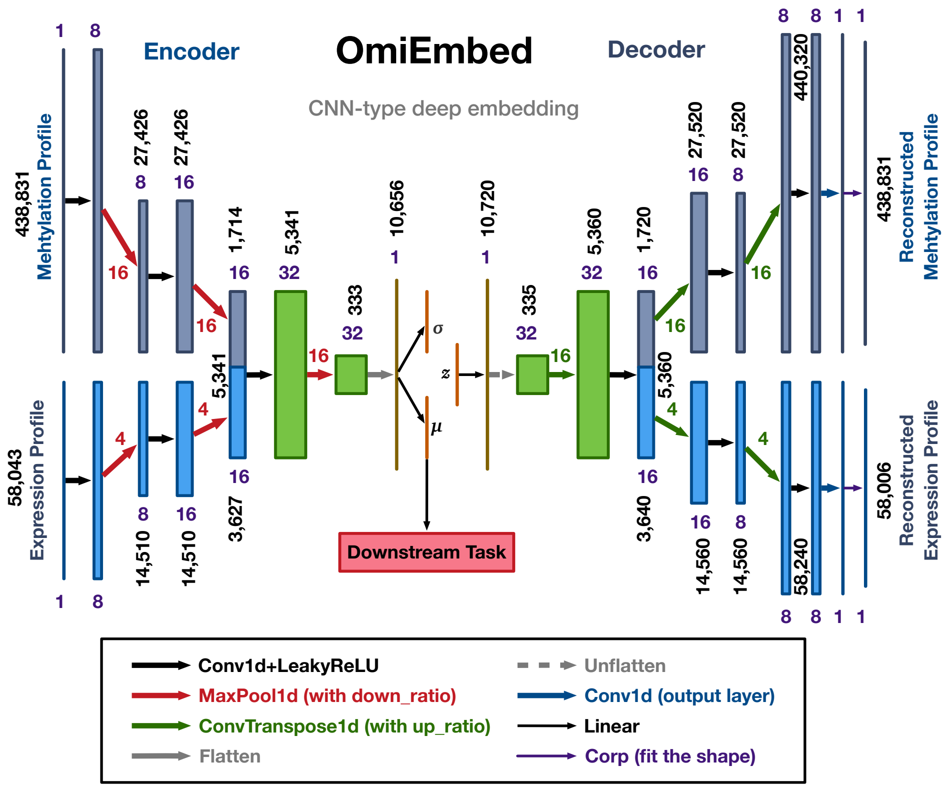

Supplementary Figure 1: The detailed network structure for the CNN-type deep embedding module in OmiEmbed. We used the integration of RNA-Seq gene expression profiles and DNA methylation profiles as an example here. The CNN-type deep embedding module can be applied for any omics type combination.

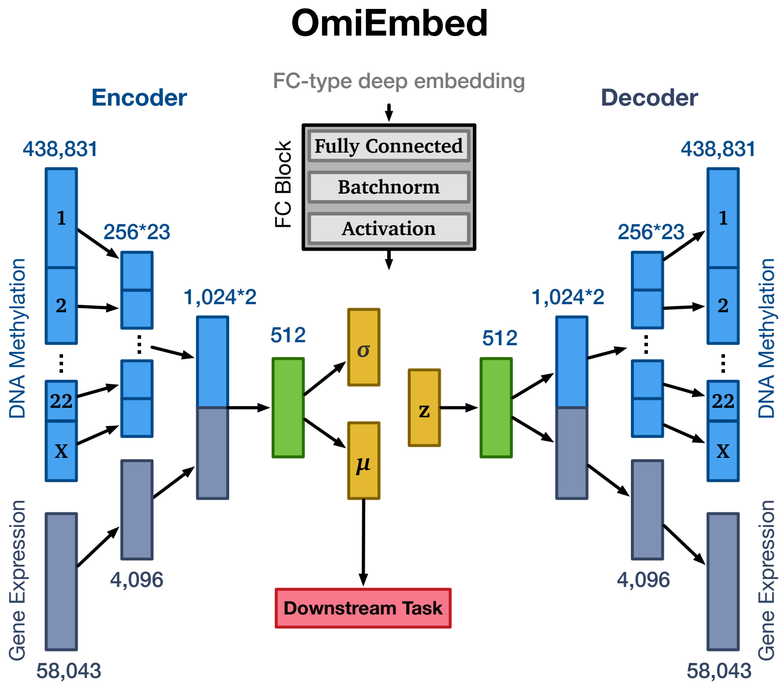

Supplementary Figure 2: The detailed network structure for the FC-type deep embedding module in OmiEmbed. CpG sites in DNA methylation profiles were separated into different FC blocks according to their targeting chromosomes to reduce the number of parameters, prevent overfitting and save the GPU memory. The chromosome separation step would be automatically processed in OmiEmbed with a built-in DNA methylation annotation if the FC-type embedding was selected. We used the integration of RNA-Seq gene expression profiles and DNA methylation profiles as an example here. The FC-type deep embedding module can be applied for any omics type combination.

Supplementary Table 1: Tumour type information of the GDC pan-cancer dataset. Sample numbers of each tumour type and the normal control for RNA-Seq gene expression profiling (A), DNA methylation profiling (B), miRNA expression profiling (C) and multi-omics profiling (M) are shown in corresponding columns.

Tumour Type	Abbr.	Project	Count A	Count B	Count C	Count M
Breast invasive carcinoma	BRCA	TCGA	1104	794	1098	773
Brain lower grade glioma	LGG	TCGA	529	534	530	525
Thyroid carcinoma	THCA	TCGA	510	515	514	509
Head and neck squamous cell carcinoma	HNSC	TCGA	502	530	525	497
Prostate adenocarcinoma	PRAD	TCGA	499	503	499	495
Lung adenocarcinoma	LUAD	TCGA	526	471	518	456
Skin cutaneous melanoma	SKCM	TCGA	471	473	450	449
Uterine corpus endometrial carcinoma	UCEC	TCGA	548	436	542	431
Bladder urothelial carcinoma	BLCA	TCGA	411	416	413	408
Liver hepatocellular carcinoma	LIHC	TCGA	374	380	375	370
Lung squamous cell carcinoma	LUSC	TCGA	501	370	478	365
Stomach adenocarcinoma	STAD	TCGA	375	395	436	335
Kidney renal clear cell carcinoma	KIRC	TCGA	535	323	521	321
Cervical squamous cell carcinoma and endocervical adenocarcinoma	CESC	TCGA	306	309	309	306
Colon adenocarcinoma	COAD	TCGA	471	309	453	303
Kidney renal papillary cell carcinoma	KIRP	TCGA	289	276	292	274
Sarcoma	SARC	TCGA	263	265	263	261
Pheochromocytoma and paraganglioma	PCPG	TCGA	183	184	184	183
Pancreatic adenocarcinoma	PAAD	TCGA	178	185	179	178
Esophageal carcinoma	ESCA	TCGA	162	186	185	162
Testicular germ cell tumours	TGCT	TCGA	156	156	156	156
Thymoma	THYM	TCGA	119	124	124	119
Acute myeloid leukemia	LAML	TCGA	151	140	188	100
Rectum adenocarcinoma	READ	TCGA	167	99	162	99
Mesothelioma	MESO	TCGA	86	87	87	86
Uveal melanoma	UVM	TCGA	80	80	80	80
Adrenocortical carcinoma	ACC	TCGA	79	80	80	79
Kidney chromophobe	KICH	TCGA	65	66	66	65
Uterine carcinosarcoma	UCS	TCGA	56	57	57	56
Lymphoid neoplasm diffuse large B-cell lymphoma	DLBC	TCGA	48	48	47	47
Cholangiocarcinoma	CHOL	TCGA	36	36	36	36
Ovarian serous cystadenocarcinoma	OV	TCGA	379	10	498	7
Glioblastoma multiforme	GBM	TCGA	168	153	0	0
Acute myeloid leukemia (paediatric)	AML	TARGET	187	0	0	0
Neuroblastoma	NBL	TARGET	157	0	0	0
High-risk Wilms tumour	WT	TARGET	126	0	0	0
Normal control			741	746	675	364
Total			11538	9736	11020	8895

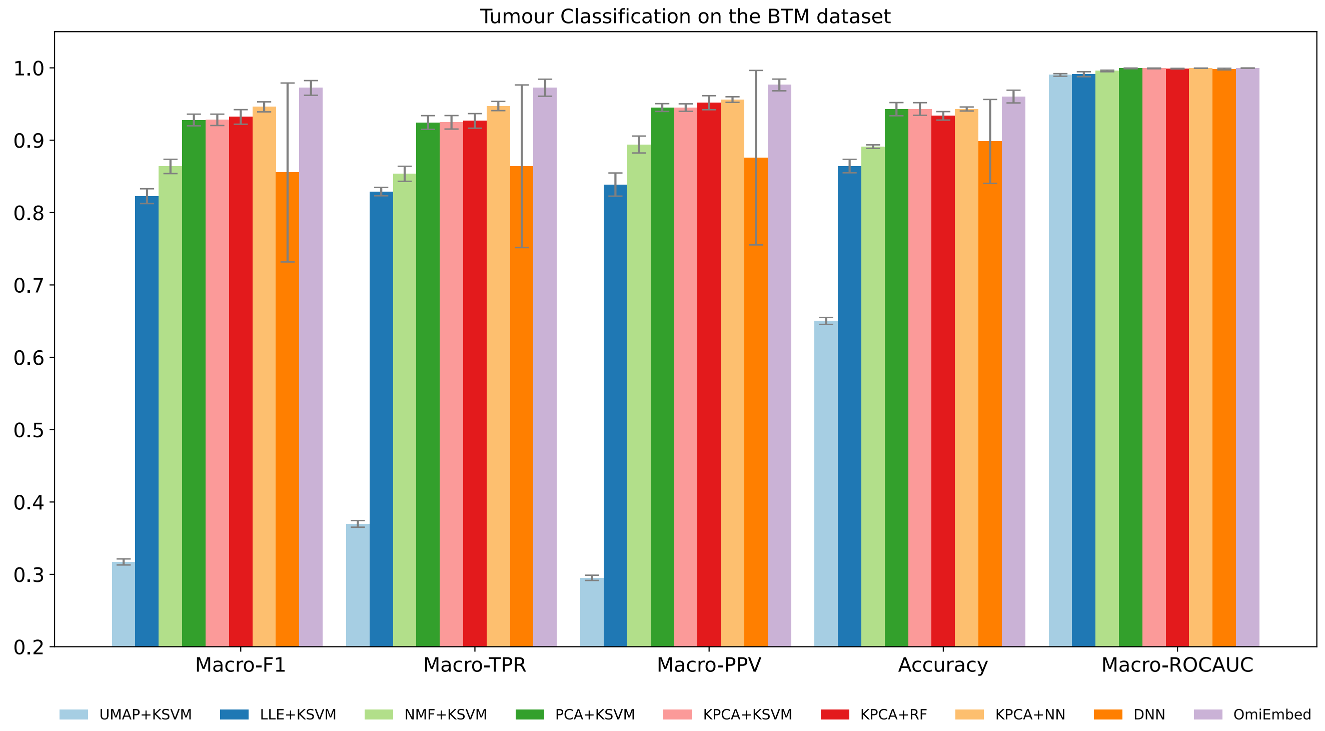

Supplementary Figure 3: Performance comparison of OmiEmbed and other eight methods for the tumour entity classification task on the BTM dataset with the methylation tumour type labels.

Supplementary Table 2: The classification performance on the BTM dataset using the methylation tumour type labels with 5-fold cross-validation, which was measured by macro-averaged F1 score (Macro-F1), macro-averaged true positive rate (Macro-TPR), macro-averaged positive predictive value (Macro-PPV), overall accuracy and macro-averaged area under the receiver operating characteristic curve (Macro-ROCAUC).

	Macro-F1	Macro-TPR	Macro-PPV	Accuracy	Macro-ROCAUC
UMAP+KSVM	0.3172±0.0042	0.3698±0.0046	0.2952±0.0036	0.6502±0.0047	0.9902±0.0018
LLE+KSVM	0.8227±0.0102	0.8290±0.0058	0.8387±0.016	0.8643±0.0093	0.9912±0.0034
NMF+KSVM	0.8637±0.0099	0.8536±0.0104	0.8940±0.0117	0.8912±0.0024	0.9957±0.0011
PCA+KSVM	0.9279±0.0081	0.9245±0.0095	0.9451±0.0054	0.9429±0.0091	0.9993±0.0004
KPCA+KSVM	0.9282±0.0079	0.9247±0.0093	0.9451±0.0052	0.9431±0.0087	0.9993±0.0004
KPCA+RF	0.9322±0.0101	0.9267±0.0101	0.9518±0.0097	0.9337±0.0059	0.9989±0.0004
KPCA+NN	0.9461±0.0069	0.9472±0.0064	0.9562±0.0039	0.9431±0.0028	0.9995±0.0002
DNN	0.8554±0.1236	0.8640±0.1124	0.8759±0.1205	0.8983±0.058	0.9984±0.0011
**OmiEmbed**	**0.9723±0.0101**	**0.9726±0.0118**	**0.9764±0.0081**	**0.9603±0.0088**	**0.9996±0.0002**

Supplementary Table 3: Detailed information for the categorical features predicted by OmiEmbed on the GDC dataset.

	Number	Label Examples
Tumour type	37	BRCA, UCEC, KIRC, LGG, LUAD, THCA, HNSC, LUSC, PRAD, Normal control, etc.
Disease stage	7	Primary tumour, Metastatic tumour, Recurrent tumor, Normal control, etc.
Primary site	29	Breast, Kidney, Lung, Brain, Colorectal, Uterus, Thyroid, Prostate, etc.
Race	6	White, Black or African American, Asian, American Indian or Alaska native, etc.
Gender	2	Male, Female

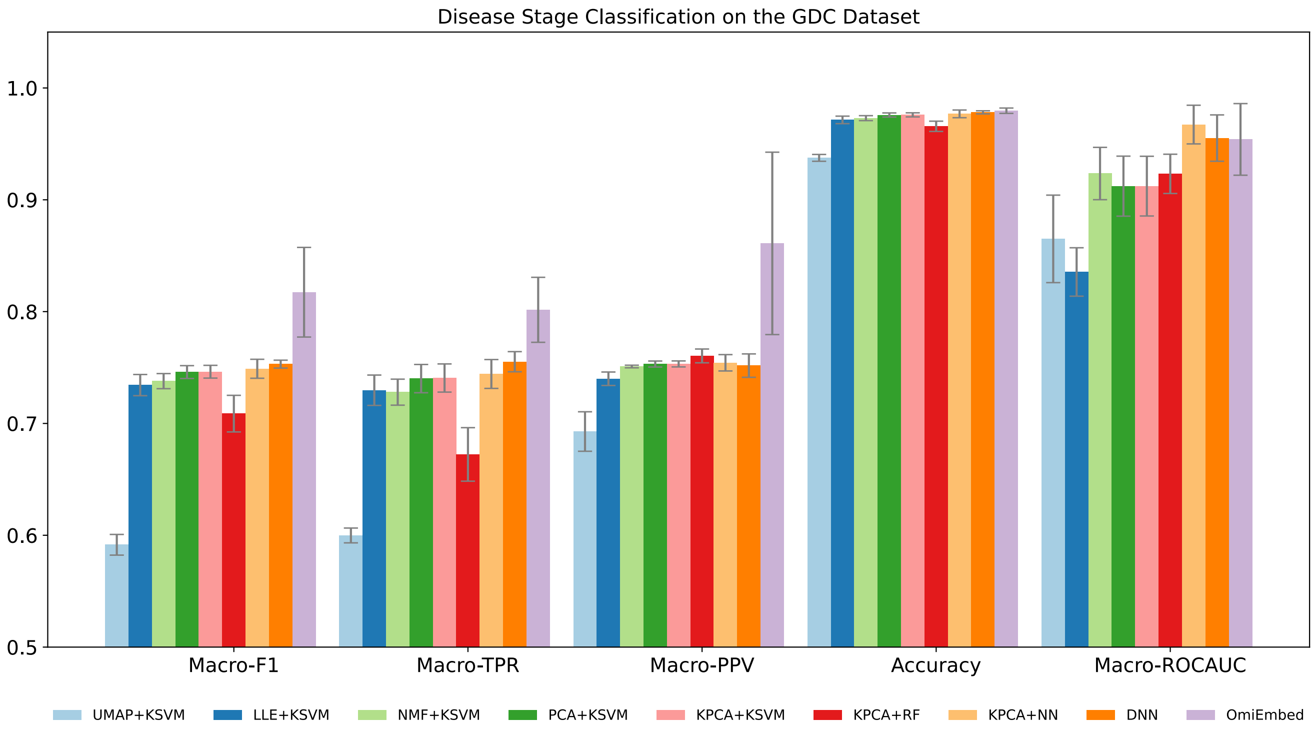

Supplementary Figure 4: Performance comparison of OmiEmbed and other eight methods for the disease stage classification task on the GDC dataset.

Supplementary Table 4: The disease stage classification performance of OmiEmbed and eight other methods on the GDC dataset.

	Macro-F1	Macro-TPR	Macro-PPV	Accuracy	Macro-ROCAUC
UMAP+KSVM	0.5915±0.0093	0.5998±0.0066	0.6928±0.0177	0.9375±0.0030	0.8650±0.0391
LLE+KSVM	0.7343±0.0095	0.7297±0.0136	0.7400±0.0060	0.9714±0.0034	0.8355±0.0216
NMF+KSVM	0.7378±0.0067	0.7280±0.0116	0.7509±0.0012	0.9730±0.0022	0.9235±0.0234
PCA+KSVM	0.7459±0.0057	0.7401±0.0126	0.7531±0.0027	0.9758±0.0018	0.9122±0.0268
KPCA+KSVM	0.7463±0.0057	0.7406±0.0126	0.7532±0.0027	0.9759±0.0018	0.9122±0.0267
KPCA+RF	0.7088±0.0164	0.6723±0.0239	0.7604±0.0062	0.9657±0.0046	0.9232±0.0176
KPCA+NN	0.7489±0.0085	0.7442±0.0129	0.7542±0.0073	0.9768±0.0034	0.9673±0.0173
DNN	0.7530±0.0035	0.7552±0.0090	0.7517±0.0105	0.9782±0.0014	**0.9552±0.0207**
**OmiEmbed**	**0.8173±0.0401**	**0.8016±0.0291**	**0.8610±0.0816**	**0.9797±0.0024**	0.9540±0.0320

Supplementary Table 5: The primary site classification performance of OmiEmbed and eight other methods on the GDC dataset.

	Macro-F1	Macro-TPR	Macro-PPV	Accuracy	Macro-ROCAUC
UMAP+KSVM	0.8200±0.0018	0.8369±0.0031	0.8268±0.0176	0.9110±0.0010	0.9914±0.0007
LLE+KSVM	0.9345±0.0084	0.9316±0.0075	0.9427±0.0114	0.9476±0.0036	0.9930±0.0021
NMF+KSVM	0.8960±0.0085	0.8892±0.0080	0.9129±0.0090	0.9517±0.0033	0.9964±0.0009
PCA+KSVM	0.9396±0.0134	0.9355±0.0131	0.9476±0.0155	0.9650±0.0041	0.9982±0.0004
KPCA+KSVM	0.9395±0.0138	0.9353±0.0137	0.9476±0.0156	0.9649±0.0041	0.9982±0.0004
KPCA+RF	0.8955±0.0050	0.8887±0.0045	0.9114±0.0089	0.9518±0.0030	0.9968±0.0012
KPCA+NN	0.9418±0.0062	0.9393±0.0071	0.9483±0.0057	0.9632±0.0027	0.9982±0.0005
DNN	0.9639±0.0066	0.9638±0.011	0.9576±0.0123	0.9593±0.0116	0.9987±0.0006
**OmiEmbed**	**0.9717±0.0066**	**0.9711±0.0046**	**0.9734±0.0095**	**0.9812±0.0023**	**0.9994±0.0003**

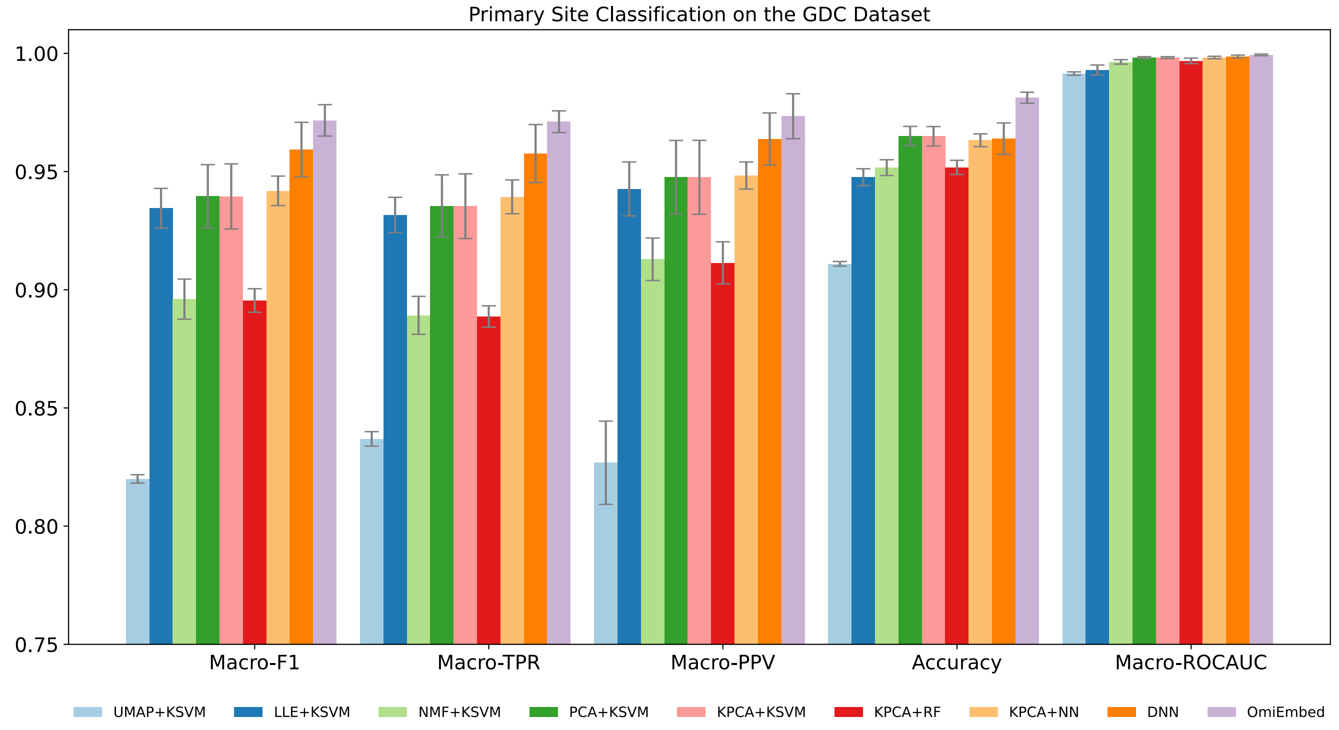

Supplementary Figure 5: Performance comparison of OmiEmbed and other eight methods for the primary site classification task on the GDC dataset.

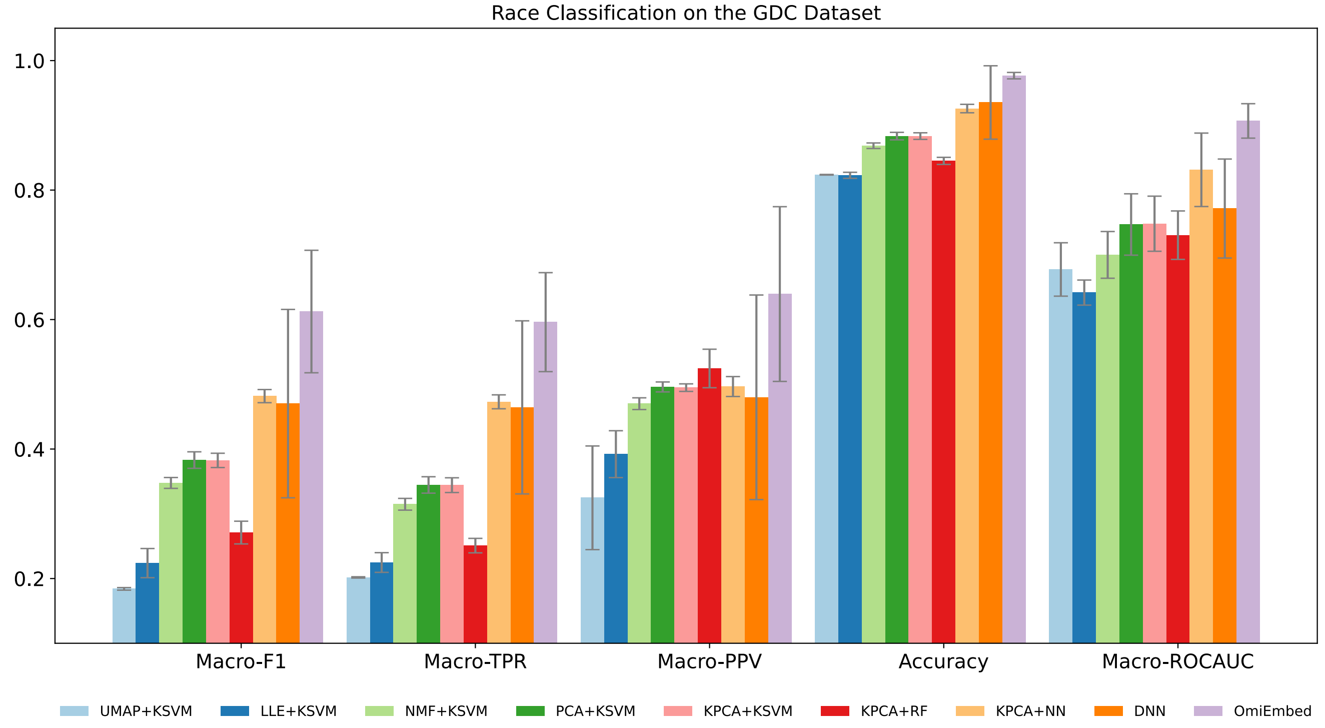

Supplementary Figure 6: Performance comparison of OmiEmbed and other eight methods for the race classification task on the GDC dataset.

Supplementary Table 6: The race classification performance of OmiEmbed and eight other methods on the GDC dataset.

	Macro-F1	Macro-TPR	Macro-PPV	Accuracy	Macro-ROCAUC
UMAP+KSVM	0.1840±0.0020	0.2017±0.0010	0.3247±0.0801	0.8237±0.0005	0.6774±0.0412
LLE+KSVM	0.2238±0.0225	0.2247±0.0152	0.3921±0.0362	0.8228±0.0047	0.6417±0.0194
NMF+KSVM	0.3476±0.0084	0.3147±0.0091	0.4700±0.0091	0.8685±0.0043	0.6999±0.0361
PCA+KSVM	0.3830±0.0128	0.3445±0.0127	0.4959±0.0077	0.8834±0.0058	0.7469±0.0474
KPCA+KSVM	0.3825±0.0110	0.3442±0.0114	0.4949±0.0058	0.8834±0.0051	0.7480±0.0426
KPCA+RF	0.2710±0.0176	0.2508±0.0112	0.5244±0.0297	0.8451±0.0055	0.7303±0.0374
KPCA+NN	0.4817±0.0101	0.4730±0.0108	0.4965±0.0154	0.9259±0.0065	0.8313±0.0567
DNN	0.4702±0.1455	0.4644±0.1337	0.4799±0.158	0.9353±0.0567	0.7715±0.0765
**OmiEmbed**	**0.6124±0.0946**	**0.5960±0.0764**	**0.6394±0.1349**	**0.9767±0.0050**	**0.9068±0.0266**

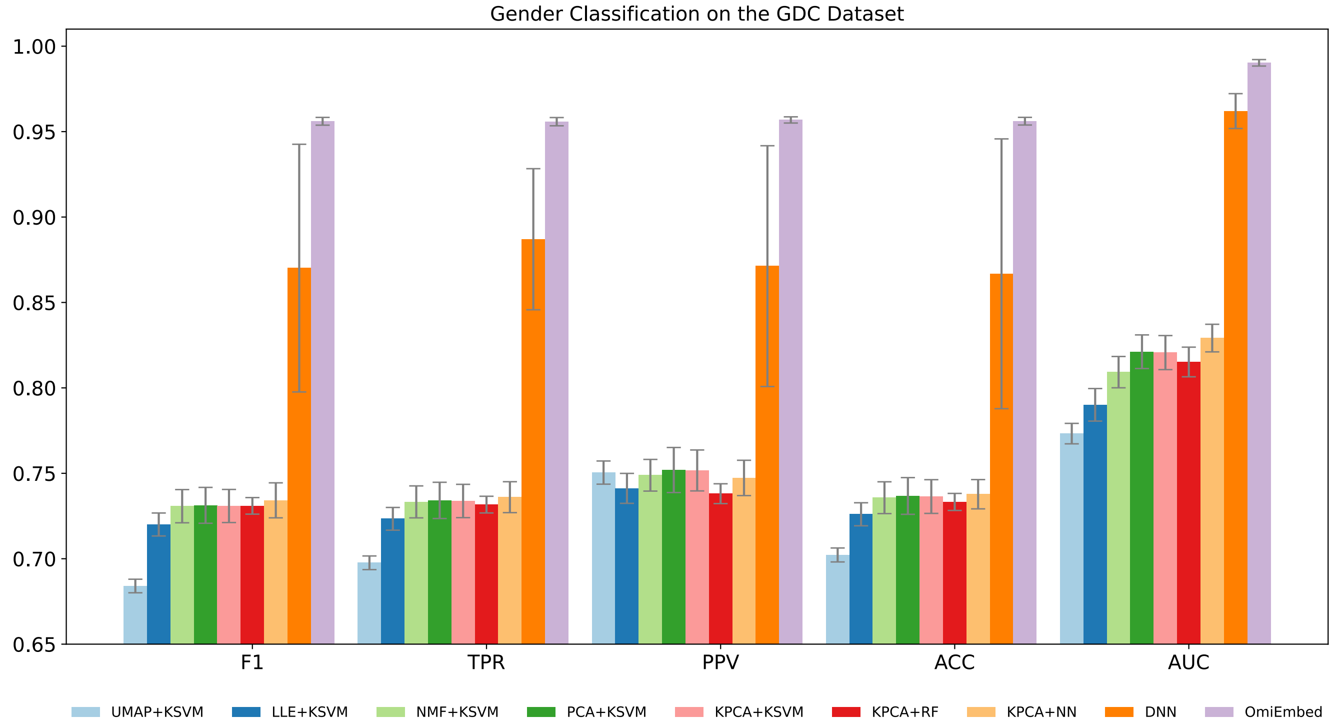

Supplementary Figure 7: Performance comparison of OmiEmbed and other eight methods for the gender classification task on the GDC dataset.

Supplementary Table 7: The gender classification performance of OmiEmbed and eight other methods on the GDC dataset.

	F1	TPR	PPV	Accuracy	ROCAUC
UMAP+KSVM	0.6840±0.0040	0.6976±0.0040	0.7504±0.0068	0.7022±0.0041	0.7732±0.0060
LLE+KSVM	0.7201±0.0067	0.7234±0.0066	0.7412±0.0088	0.7260±0.0067	0.7901±0.0095
NMF+KSVM	0.7308±0.0097	0.7333±0.0094	0.7489±0.0093	0.7357±0.0093	0.8092±0.0092
PCA+KSVM	0.7313±0.0105	0.7341±0.0106	0.7519±0.0132	0.7367±0.0107	0.8212±0.0098
KPCA+KSVM	0.7309±0.0097	0.7338±0.0098	0.7517±0.0120	0.7364±0.0099	0.8207±0.0100
KPCA+RF	0.7309±0.0048	0.7317±0.0049	0.7381±0.0058	0.7332±0.0050	0.8152±0.0087
KPCA+NN	0.7342±0.0102	0.7360±0.0091	0.7473±0.0103	0.7378±0.0086	0.8292±0.0081
DNN	0.8701±0.0725	0.887±0.0413	0.8713±0.0705	0.8668±0.079	0.962±0.0102
**OmiEmbed**	**0.9560±0.0023**	**0.9558±0.0024**	**0.9568±0.0018**	**0.9561±0.0022**	**0.9903±0.0019**

Supplementary Table 8: Detailed tumour type information of the BTM dataset with the methylation class labels defined by the original paper.

Methylation Class	Abbr.	Count
Embryonal tumor with multilayered rosettes	ETMR	49
Medulloblastoma, WNT	MB, WNT	48
Medulloblastoma, subclass group 3	MB, G3	99
Medulloblastoma, subclass group 4	MB, G4	181
Medulloblastoma, subclass SHH A (children and adult)	MB, SHH CHL AD	126
Medulloblastoma, subclass SHH B (infant)	MB, SHH INF	65
Atypical teratoid/rhabdoid tumor, subclass MYC	ATRT, MYC	31
Atypical teratoid/rhabdoid tumor, subclass SHH	ATRT, SHH	51
Atypical teratoid/rhabdoid tumor, subclass TYR	ATRT, TYR	39
CNS neuroblastoma with FOXR2 activation	CNS NB, FOXR2	43
CNS high grade neuroepithelial tumor with BCOR alteration	HGNET, BCOR	26
Diffuse midline glioma H3 K27M mutant	DMG, K27	117
Glioblastoma, IDH wildtype, H3.3 G34 mutant	GBM, G34	54
Glioblastoma, IDH wildtype, subclass mesenchymal	GBM, MES	160
Glioblastoma, IDH wildtype, subclass RTK I	GBM, RTK I	108
Glioblastoma, IDH wildtype, subclass RTK II	GBM, RTK II	261
Glioblastoma, IDH wildtype, subclass RTK III	GBM, RTK III	22
Glioblastoma, IDH wildtype, subclass midline	GBM, MID	33
Glioblastoma, IDH wildtype, subclass MYCN	GBM, MYCN	33
Central neurocytoma	CN	23
Diffuse leptomeningeal glioneuronal tumor	DLGNT	12
Cerebellar liponeurocytoma	LIPN	11
Low grade glioma, desmoplastic infantile astrocytoma / ganglioglioma	LGG, DIG/DIA	8
Low grade glioma, dysembryoplastic neuroepithelial tumor	LGG, DNT	56
Low grade glioma, rosette forming glioneuronal tumor	LGG, RGNT	12
Retinoblastoma	RETB	19
Esthesioneuroblastoma, subclass A	ENB, A	24
Esthesioneuroblastoma, subclass B	ENB, B	16
Paraganglioma, spinal non-CIMP	PGG, nC	20
Low grade glioma, ganglioglioma	LGG, GG	26
Craniopharyngioma, adamantinomatous	CPH, ADM	25
Craniopharyngioma, papillary	CPH, PAP	20
Pituitary adenoma, ACTH	PITAD, ACTH	19
Pituitary adenoma, FSH/LH	PITAD, FSH LH	23
Pituitary adenoma, prolactin	PITAD, PRL	8
Pituitary adenoma, STH densely granulated, group A	PITAD, STH DNS A	9
Pituitary adenoma, STH densely granulated, group B	PITAD, STH DNS B	13
Pituitary adenoma, STH sparsely granulated	PITAD, STH SPA	17
Pituitary adenoma, TSH	PITAD, TSH	11
Pituicytoma / granular cell tumor / spindle cell oncocytoma	PITUI	30
Ependymoma, myxopapillary	EPN, MPE	45
Ependymoma, posterior fossa group A	EPN, PF A	127
Ependymoma, posterior fossa group B	EPN, PF B	59
Ependymoma, RELA fusion	EPN, RELA	90
Ependymoma, spinal	EPN, SPINE	34
Ependymoma, YAP fusion	EPN, YAP	11
Subependymoma, posterior fossa	SUBEPN, PF	40
Subependymoma, spinal	SUBEPN, SPINE	12
Subependymoma, supratentorial	SUBEPN, ST	20
Chordoid glioma of the third ventricle	CHGL	12

Low grade glioma, subependymal giant cell astrocytoma	LGG, SEGA	22
Low grade glioma, subclass hemispheric pilocytic astrocytoma and ganglioglioma	LGG, PA MID	55
Low grade glioma, subclass midline pilocytic astrocytoma	LGG, PA PF	154
Anaplastic pilocytic astrocytoma	ANA PA	46
CNS high grade neuroepithelial tumor with MN1 alteration	HGNET, MN1	26
Infantile hemispheric glioma	IHG	15
Low grade glioma, MYB/MYBL1	LGG, MYB	27
Low grade glioma, subclass posterior fossa pilocytic astrocytoma	LGG, PA/GG ST	45
(Anaplastic) pleomorphic xanthoastrocytoma	PXA	67
Schwannoma	SCHW	31
Melanotic schwannoma	SCHW, MEL	12
Papillary tumor of the pineal region group A	PTPR, A	9
Papillary tumor of the pineal region group B	PTPR, B	23
Pineoblastoma group A / intracranial retinoblastoma	PIN T, PB A	9
Pineoblastoma group B	PIN T, PB B	23
Pineal parenchymal tumor	PIN T, PPT	20
Chordoma	CHORDM	11
Ewing sarcoma	EWS	17
Hemangioblastoma	HMB	27
Meningioma	MNG	149
Solitary fibrous tumor / hemangiopericytoma	SFT HMPC	18
CNS Ewing sarcoma family tumor with CIC alteration	EFT, CIC	13
Melanoma	MELAN	18
Melanocytoma	MELCYT	19
Plexus tumor, subclass adult	PLEX, AD	23
Plexus tumor, subclass paediatric A	PLEX, PED A	16
Plexus tumor, subclass paediatric B	PLEX, PED B	49
IDH glioma, subclass astrocytoma	A IDH	172
IDH glioma, subclass high grade astrocytoma	A IDH, HG	87
IDH glioma, subclass 1p/19q codeleted oligodendroglioma	O IDH	163
Lymphoma	LYMPHO	14
Plasmacytoma	PLASMA	8
Control tissue, pituitary gland anterior lobe	CONTR, ADENOPIT	9
Control tissue, cerebellar hemisphere	CONTR, CEBM	8
Control tissue, hemispheric cortex	CONTR, HEMI	13
Control tissue, hypothalamus	CONTR, HYPTHAL	9
Control tissue, inflammatory tumor microenvironment	CONTR, INFLAM	24
Control tissue, pineal gland	CONTR, PINEAL	12
Control tissue, pons	CONTR, PONS	12
Control tissue, reactive tumor microenvironment	CONTR, REACT	23
Control tissue, white matter	CONTR, WM	9
**Total**		**3905**

Supplementary Table 9: Detailed tumour type information of the BTM dataset with the pathological class labels defined by the 2016 WHO classification of CNS tumours.

Class ID	Pathological Class	count
0	Glioblastoma, IDH-wildtype	686
1	Pilocytic astrocytoma	235
2	Medulloblastoma, genetically defined, SHH-activated	189
3	Medulloblastoma, genetically defined, group 4	180

4	Anaplastic ependymoma	145
5	Ependymoma	142
6	Anaplastic astrocytoma, IDH-mutant	125
7	Atypical teratoid/rhabdoid tumour	121
8	Diffuse midline glioma, H3 K27M-mutant	115
9	Meningioma	105
10	Anaplastic oligodendroglioma, IDH-mutant and 1p/19q-codeleted	97
11	Medulloblastoma, genetically defined, group 3	96
12	CNS embryonal tumour, NOS	91
13	Diffuse astrocytoma, IDH-mutant	82
14	Ependymoma, RELA fusion-positive	74
15	Oligodendroglioma, IDH-mutant and 1p/19q-codeleted	73
16	Dysembryoplastic neuroepithelial tumour	60
17	Subependymoma	52
18	Embryonal tumour with multilayered rosettes, C19MC-altered	49
19	Medulloblastoma, genetically defined, WNT-activated	47
20	Ganglioglioma	45
21	Glioblastoma, IDH-mutant	44
22	Esthesioneuroblastoma/Olfactory neuroblastoma	40
23	Myxopapillary ependymoma	39
24	Pineoblastoma	35
25	Atypical choroid plexus papilloma	33
26	Pleomorphic xanthoastrocytoma	32
27	Papillary tumour of the pineal region	32
28	Schwannoma	30
29	Choroid plexus carcinoma	30
30	Haemangioblastoma	27
31	Atypical meningioma	27
32	Gliosarcoma, IDH-wildtype	25
33	Choroid plexus papilloma	25
34	Adamantinomatous craniopharyngioma	25
35	Low tumour cell content Glioblastoma, IDH wildtype with high inflammatory cell infiltration	24
36	Pituitary adenoma gonadotropin producing	23
37	Retinoblastoma	22
38	Central neurocytoma	22
39	Subependymal giant cell astrocytoma	21
40	Pituitary adenoma densely granulated GH/STH producing	21
41	Paraganglioma	21
42	Anaplastic pleomorphic xanthoastrocytoma	21
43	Papillary craniopharyngioma	20
44	Anaplastic pilocytic astrocytoma	20
45	Pituitary adenoma ACTH producing	19
46	Melanocytoma	19
47	Solitary fibrous tumour / haemangiopericytoma	18
48	Pituitary adenoma sparsely granulated GH/STH producing	18
49	Ewing sarcoma / peripheral primitive neuroectodermal tumour	16
50	Malignant melanoma	16
51	Pineal parenchymal tumour of intermediate differentiation	14
52	Anaplastic pilocytic astrocytoma (unresolved status)	14
53	Diffuse large B cell lymphoma (DLBCL)	14
54	Anaplastic astrocytoma, IDH-wildtype	13
55	Normal cortex	13
56	Chordoid glioma of the third ventricle	12
57	Angiocentric glioma	12
58	Normal Pons	12

59	Normal pineal gland	12
60	Cerebellar liponeurocytoma	11
61	Rosette-forming glioneuronal tumour	11
62	Anaplastic (malignant) meningioma	11
63	Chordoma	11
64	Pituitary adenoma TSH producing	11
65	Pituicytoma	11
66	Melanotic schwannoma	10
67	Granular cell tumour	10
68	Diffuse leptomeningeal glioneuronal tumour	10
69	Normal corpus callosum	9
70	Spindle cell oncocytoma	9
71	Normal hypothalamus	9
72	Astroblastoma	9
73	Normal pituitary anterior lobe	8
74	Desmoplastic infantile astrocytoma and ganglioglioma	8
75	Normal cerebellum cortex	8
76	Pituitary adenoma prolactin producing	8
77	Pilomyxoid astrocytoma	8
78	Plasmacytoma	8
79	Anaplastic ganglioglioma	5
80	Chordoid meningioma	4
81	Low grade glioma with MYB alteration (no official WHO diagnosis)	4
82	Diffuse astrocytoma, IDH-wildtype	3
83	Paediatric diffuse astrocytoma (unresolved status)	3
84	Infantile hemispheric glioma (no official WHO diagnosis)	3
85	Medulloblastoma, NOS	3
86	CNS high-grade neuroepithelial tumour with MN1 alteration (no official WHO diagnosis)	2
87	Pilocytic astrocytoma, pilomyxoid variant	2
88	Normal pituitary anterior lobe (fetal tissue, gestation week 20)	1
89	Dysplastic gangliocytoma of cerebellum (Lhermitte-Duclos)	1
90	Gangliocytoma	1
91	CNS high-grade neuroepithelial tumour with BCOR alteration (no official WHO diagnosis)	1
92	Tanycytic ependymoma	1
93	Rhabdoid meningioma	1
**Total**		**3905**
